# Efficient Non‐Invasive Rejuvenation of Spent Lithium Iron Phosphate Batteries Through Controlled Overdischarge

**DOI:** 10.1002/adma.202522927

**Published:** 2026-02-10

**Authors:** Jinu Song, Yujie Chen, Nianji Zhang, Cancan Peng, Huan Li, Chao Ye, Shi‐Zhang Qiao

**Affiliations:** ^1^ School of Chemical Engineering The University of Adelaide Adelaide South Australia Australia

**Keywords:** Cu dissolution, LFP recycling, non‐invasive rejuvenation, overdischarge

## Abstract

Recycling lithium iron phosphate (LFP) batteries presents critical economic and environmental challenges because of their low metal value and high energy intensity of conventional metallurgical processes. While direct recycling methods offer a pathway for lithium replenishment, they are often hindered by stringent impurity controls and complex operating conditions that limit scalability. Here, we introduce a controlled overdischarge (COD) protocol as a non‐invasive strategy to rejuvenate spent LFP (S‐LFP) batteries. COD selectively decomposes the solid‐electrolyte interphase, releasing trapped Li^+^ and reducing Li/Fe antisite defects while simultaneously suppressing copper dissolution. The COD protocol recovers 9.56% of lost capacity and extends lifespan by over 200 cycles. Furthermore, compared to metallurgical recycling, this method markedly lowers greenhouse gas emissions to 168 g kg^−1^ and energy consumption to 3 MJ kg^−1^ of feedstock. These findings highlight COD as a sustainable and scalable alternative for S‐LFP battery recycling.

## Graphical abstract

This strategy rejuvenates spent lithium iron phosphate battery by non‐invasively targeting Li^+^ trapped within the solid‐electrolyte interphase (SEI). This method mitigates copper dissolution and reduces Li/Fe antisite defects, achieving 9.56% capacity recovery and 214 cycles lifespan extension. The process requires only 3 MJ kg^−1^ of energy and emitting 168 g kg^−1^ of greenhouse gases per feedstock.

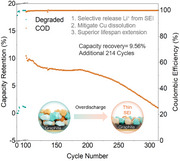

## 1 Introduction

The global deployment of lithium iron phosphate (LFP) batteries has undergone explosive growth in recent years, driven by their high safety, long cycle life, and low cost [1, 2, 3, 4]. This widespread adoption is generating a rapidly growing volume of spent LFP (S‐LFP) batteries, posing serious environmental and resource recovery challenges [5]. Unlike other cathode materials, LFP inherently contains low levels of valuable metals. This renders conventional metallurgical recycling economically unattractive and environmentally challenging due to the high energy input and extensive chemical processing required [3, 6]. To address these drawbacks, direct recycling has emerged as a promising approach for S‐LFP batteries. This strategy aims to regenerate electrode materials while preserving their original structure and avoiding the chemical‐ and energy‐ intensive metallurgical processes. However, direct recycling processes that begin with the “black mass” require impurity control and specific conditions, thus limiting their sustainability in practical applications [7, 8, 9, 10, 11, 12, 13, 14].

To further increase the sustainability of S‐LFP recycling, researchers have explored rejuvenation strategies that avoid fully disassembling the batteries. Such non‐disassemble methods employ pre‐modified electrolytes to reactivate deactivated lithium or supplement active lithium through reversible redox reactions [15, 16]. While promising, these approaches can introduce physical damage through reagent injections. More recently, machine‐learning‐guided design of lithium salts has drawn attention to achieve high recovery rates [17]. Yet the cost of preparing these lithiation reagents may be a practical obstacle. Overall, there is an urgent need for simpler non‐invasive rejuvenation strategies that operate on intact cells and offer a practical, cost‐effective, and scalable pathway to extend the lifespan of S‐LFP batteries.

**FIGURE 1:**
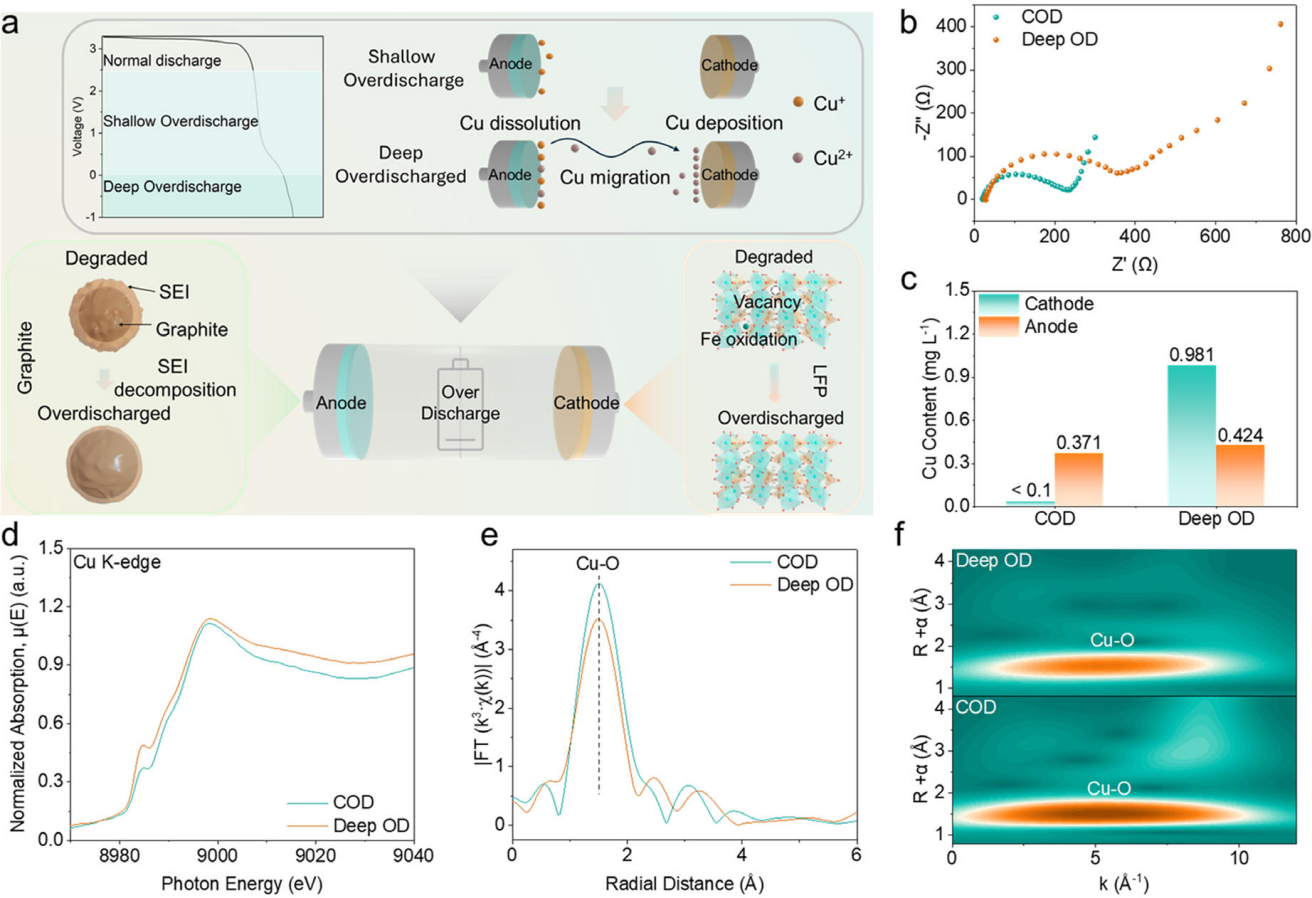
Study of overdischarge in the battery system. (a) Schematic illustration for overdischarge mechanism; (b) EIS spectra collected at COD and Deep OD points; (c) Cu concentration in COD and Deep OD graphite and LFP samples based on ICP‐MS; (d) The normalized Cu K‐edge XANES spectra, (e) EXAFS spectra (R space plots), and (f) Wavelet transform of the EXAFS spectra of COD and Deep OD graphite samples.

Regulating external physical fields such as voltage and temperature represents a promising non‐invasive strategy to rejuvenate S‐LFP batteries without chemical additives [18, 19, 20]. For instance, utilizing a deep discharge below 0 V was demonstrated to extend battery lifespan by refining the solid electrolyte interphase (SEI). Despite this promise, lifespan extension via deep discharge has been limited to the pretreatment process for battery recycling due to insufficiently understood underlying mechanisms [21, 22]. In practice, as shown in Figure 1a, overdischarge is generally defined as lowering a cell's voltage below 2.5 V. It is conventionally regarded as a critical degradation protocol because it repeatedly damages the SEI and consumes lithium during subsequent cycling, thereby accelerating capacity fade [23, 24, 25]. At extreme depth, overdischarge can induce severe side reactions such as copper (Cu) current collector dissolution, gas evolution, dendrite formation, separator damage, and internal short circuit [25, 26, 27, 28, 29, 30, 31]. Therefore, a systematic understanding of how side reactions depend on overdischarge voltage is essential for safe and practical application.

In this study, we introduce controlled overdischarge (COD under 0.5 V cutoff) as a non‐invasive strategy to rejuvenate spent LFP batteries. Unlike deep overdischarge (Deep OD, −0.25 V cutoff), which accelerates Cu dissolution and irreversible reactions. Under COD, inactive Li^+^ is extracted from the SEI and inserted into the cathode, reducing Li/Fe antisite defects while mitigating side reactions consistent with the 9.56% capacity recovery and extending lifespan by 214 cycles without disassembly or chemical additives.

## 2 Results and Discussion

### 2.1 Stepwise Mechanistic Insight During Overdischarge

We systematically investigated overdischarge using full cell with LFP cathode and graphite anode reassembled from degraded 18650 cell with 80% capacity retention of original capacity. Unique plateau with increasing the depth of overdischarge and the corresponding dQ/dV peak indicated unspecified chemical reactions inside the battery (Figure S1). To understand how these trends manifest in the internal battery environment, electrochemical impedance spectroscopy (EIS) was performed immediately after overdischarge (Figure 1b; Figure S2a). Compared with the normal discharge condition, the semicircle corresponding to both electrodes increased progressively with increasing overdischarge depth, indicating the accumulation of byproducts from SEI/cathode electrolyte interphase (CEI) decomposition, electrolyte decomposition, electrode particle damage and Cu dissolution [26, 32, 33]. When the cutoff potential was lowered below 0.25 V, the semicircle rose sharply, implying severe side reactions.

Distribution of relaxation times (DRT) analysis (Figure S2b) provided further mechanistic insights. In the high to mid frequency region (10^0^–10^5^ Hz), the relaxation peak broadened and intensified with increasing overdischarge depth. This suggests that SEI/CEI decomposition dominates at the early stage above COD, whereas Cu dissolution and electrolyte decomposition become increasingly pronounced below 0.25 V. The low‐frequency region (below 10^0^ Hz) exhibits a non‐linear trend, where an initial stage at 1.0 V suggests a transient phase of surface heterogeneity caused by partial SEI decomposition. As overdischarge progresses to 0.25 and 0.01 V, the interphase stabilizes as ion‐transport pathways within the active regions become partially activated, leading to a temporary reduction in resistance. In contrast, Deep OD leads to a recurrence of severe Li^+^ diffusion limitations, driven by overdischarge‐induced side reactions that block Li^+^ pathways. These results highlight the severe kinetic degradation induced by Deep OD, ultimately accelerating capacity fading and elevating safety risks. Additional insights were gained through the attenuated total reflectance‐Fourier transform infrared (ATR‐FTIR) (Figure S3). A distinct P–F signal on the anode was observed below 0.25 V [34, 35, 36], suggesting that overdischarge not only promotes Cu dissolution but also induces electrolyte decomposition [27, 30]. Combined with SEI decomposition, these reactions accelerate SEI breakdown and intensify capacity loss in subsequent cycles. These results indicate that COD may mitigate side reactions and uncontrolled Deep OD can potentially activate irreversible degradation pathways, emphasizing the necessity of precise voltage control for safe and effective rejuvenation. The quantitative analysis was conducted to specifically verify Cu dissolution which represents one of the most critical degradation mechanisms. The anode Cu current collector dissolves into Cu species, migrates through the electrolyte, and eventually deposits on both electrodes. Such Cu deposition can cause severe issues, including anode delamination and Li dendrite accumulation on the cathode, separator, and anode surface, hindering Li^+^ transport and intercalation/deintercalation.

To probe this mechanism, we first performed inductively coupled plasma−mass spectrometry (ICP‐MS) measurements. Under COD, Cu ions were scarcely detectable on the cathode side, whereas Deep OD resulted in a dramatic increase in Cu concentration, confirming extensive Cu dissolution and migration (Figure 1c). These results demonstrate that Cu dissolution and deposition become dominant side reactions with increasing overdischarge depth. To further resolve the state of oxidation and coordination environment of the dissolved Cu ions, synchrotron‐based Cu K‐edge X‐ray absorption spectroscopy (XAS) was performed. At COD, the X‐ray absorption near edge structure (XANES) spectrum (Figure 1d) exhibited less oxidized Cu local environment. In contrast, Deep OD showed more oxidized Cu local environment by continuous dissolution of Cu ions from the anode and migration toward the cathode due to concentration gradient [32], consistent with the Cu accumulation trends detected by ICP‐MS. Fourier‐transformed extended X‐ray absorption fine structure (EXAFS) oscillations (Figure 1e) provided further structural insight. Comparable Cu─O bonding at around 1.45 Å was observed under COD. However, under Deep OD, the Cu─O signal was reduced, demonstrating more Cu dissolution and redeposition to increase structural disorder that hinder Li^+^ transport and accelerate electrolyte decomposition [33]. Complementary wavelet‐transformed of the EXAFS results (Figure 1f) reinforced this trend, revealing weaker Cu─O features with a disordered environment and evidence of more Cu dissolution. Such Cu dissolution under Deep OD increased the risk of metallic Cu and lithium dendrite growth on both electrodes and the separator [26].

Together, these results demonstrate not only a more oxidized Cu local environment under Deep OD but also a coordination environment change, highlighting the critical role of COD in suppressing Cu dissolution. Importantly, COD enables selective SEI decomposition and lithium recovery. Its controllability mitigates severe side reactions, particularly Cu dissolution, which has been a critical bottleneck in overdischarge‐based rejuvenation.

### 2.2 Electrochemical Performances After Rejuvenation by Controlled Overdischarge

**FIGURE 2:**
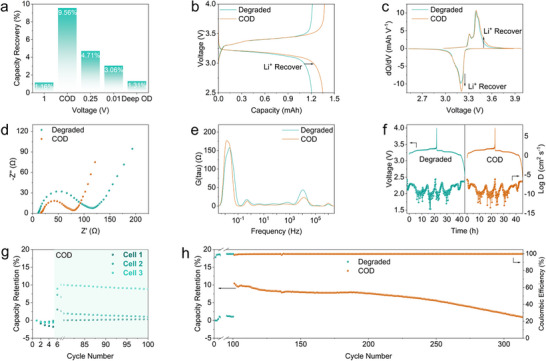
Electrochemical performance of the overdischarge strategy. (a) Capacity retention after different overdischarge conditions; (b) GCD curves of cell before and after COD at 0.3 C and c) corresponding dQ/dV profiles; (d) EIS collected before and after COD and (e) DRT spectra derived from the EIS spectra; (f) GITT profiles degraded and COD cell with corresponding diffusion coefficients; (g) Cycling performance of COD applying on multiple degraded cells at 0.3 C and (h) long‐term cycling performance of COD at 0.3 C.

This voltage‐dependent behavior of the S‐LFP cell underscores the strategic advantage of COD. As shown in Figure 2a, the cell overdischarged to 1.0 V showed a slight capacity increase of approximately 1.16%, indicating reactivation of lithium trapped in the graphite anode. When the cutoff voltage was lowered into the COD region, the capacity increased by 9.56%, reflecting additional lithium extraction from partial SEI decomposition. Overdischarging to 0.25 and 0.01 V produced smaller capacity gains of 4.71% and 3.06%, respectively, implying the onset of side reactions. Under the Deep OD, the capacity gain was comparable to that at 1.0 V, suggesting that extensive electrolyte decomposition and Cu dissolution resulted in SEI reformation and lithium consumption, thereby limiting recovery. Galvanostatic charge‐ discharge (GCD) and corresponding dQ/dV curves before and after overdischarge were analyzed (Figure 2b,c; Figures S4 and S5). Under COD, the increase in peak area and the appearance of new features in the dQ/dV profile reflect lithium reactivation, thereby rejuvenating the S‐LFP. By contrast, as overdischarge progresses into the uncontrolled Deep OD regime, the peaks shrink and shift, indicating enhanced polarization arising from excessive Cu dissolution. This behavior reflects the growth of internal resistance and accelerated degradation, underscoring the detrimental impact of Deep OD. Collectively, these observations highlight the superior cycling stability and practical promise of the COD strategy.

As shown in previous measurements, the EIS semicircle increased progressively with overdischarge depth, suggesting that the SEI/CEI decomposed and the surface became covered by byproducts. Interestingly, in the rejuvenated cell subjected to COD after formation cycling, the semicircles markedly decreased (Figure 2d; Figure S6). This indicates that COD protocol removes thick interphase layers and reduces internal resistance. In contrast, Deep OD caused larger semicircles, implying elevated resistance and serious side reactions. COD reduced the high‑frequency peak associated with the SEI/CEI, whereas increasing overdischarge depth caused the peak to vanish or shift, reflecting uncontrolled side reactions (Figure 2e; Figure S7). Collectively, these results confirm that COD refines the interphase while mitigating side reactions, whereas deep overdischarge thickens the SEI/CEI and hampers Li^+^ transport.

The galvanostatic intermittent titration technique (GITT) was conducted to investigate the COD effect on Li^+^ transport kinetics (Figure 2f). Although minor variations were observed at different states of charge, the overall Li^+^ diffusion coefficient profile remained stable after COD, indicating that the COD does not impair Li^+^ transport kinetics. This was further supported by cyclic voltammetry (CV) measurements performed at scan rates from 0.1 to 1.0 mV s^−1^ before and after COD (Figure S8). The Li^+^ diffusion coefficients determined using the Randles–Sevcik equation [37], decreased slightly from 0.77 × 10^−11^ to 0.74 × 10^−11^ cm^2^ s^−1^ and from 0.70 × 10^−11^ to 0.69 × 10^−11^ cm^2^ s^−1^, confirming that COD does not hinder Li^+^ transport (Table S1).

Long term cycling tests revealed that COD significantly extends battery lifespan (Figure 2g). When applied to multiple degraded cells, COD enabled more than 100 additional charge–discharge cycles, with a single cell achieving up to 214 cycles (Figure 2h), while maintaining stable performance. Although capacity recovery was continuously observed with the repeated COD applications (Figure S9), no additional capacity recovery was observed after the fourth application. The marked decreases in recovery efficiency and cycle life extension suggest that cumulative effects such as SEI instability and trace Cu dissolution and redeposition due to repeated overdischarge, ultimately limit the effectiveness of the COD protocol.

Building on this mechanistic insight from coin cell, we validated the COD strategy in commercial 18 650 cylindrical batteries (Figure S10). After COD treatment, the cell recovered approximately 6.46% of its capacity and sustained around 50 additional cycles. The dQ/dV analysis also confirmed lithium reactivation, as evidenced by an increase in peak area. The subsequent cycling tests confirmed further cycling extension, and repeated COD applications. Testing across multiple degraded 18 650 cells consistently demonstrated capacity recovery, highlighting the repeatability and broader applicability of COD. Properly regulated COD thus represents a practical, non‑invasive strategy to extend the lifespan of commercial S‐LFP batteries.

However, the relatively lower recovery efficiency in commercial 18 650 cells is likely attributed to their higher mass loading and structural complexity compared to coin cells. The reactivation of Li^+^ through SEI decomposition is likely less effective in the deeper regions of the thick electrodes, thereby reducing overall efficiency. These results suggest that developing more advanced COD protocols is essential for the practical application of this technology in large‐sized commercial batteries.

### 2.3 Structural Effects of Overdischarge on LFP and SEI Dynamics in Graphite

**FIGURE 3:**
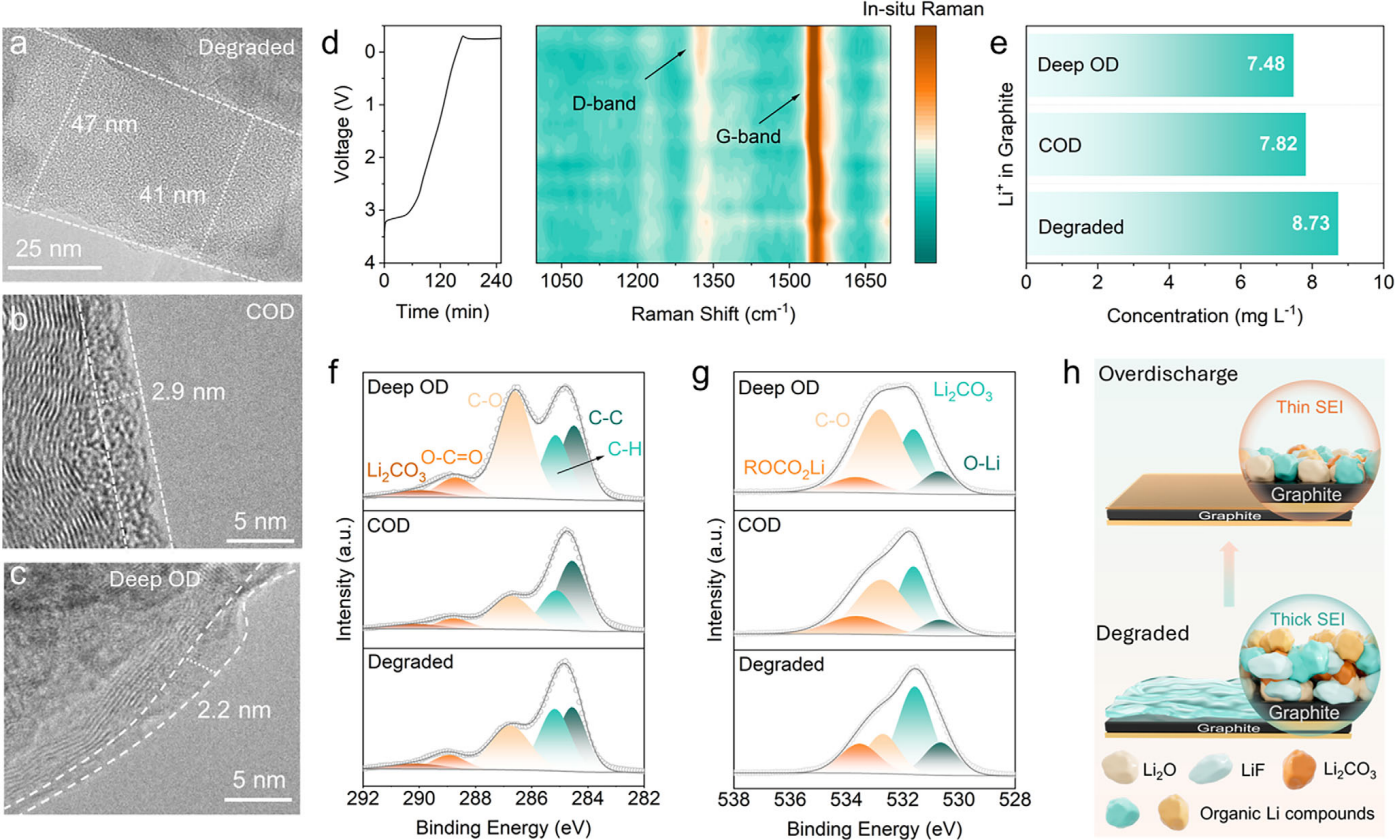
Microstructure characterization of graphite anode under different overdischarge conditions. (a–c) TEM images of SEI on graphite of Degraded, COD and Deep OD; (d) In situ Raman spectrum of graphite anode during overdischarge with galvanostatic discharging curve; (e) Li^+^ concentration in graphite of Degraded, COD and Deep OD based on ICP−MS; XPS analysis of f) C 1s and (g) O 1s for Degraded, COD and Deep OD; (h) Schematic illustration of decomposition of SEI layer from graphite surface during overdischarge.

Electrochemical tests indicated that a proper COD protocol can selectively reactivate Li species and extend cell lifespan. To further elucidate this mechanism, the anode morphology and element compositions at various discharge voltages were examined by scanning electron microscopy (SEM) (Figure S11 and Table S2). SEM images show no significant particle damage from overdischarge above COD, yet distinct surface deposits appeared below 0.25 V. Energy‐dispersive X‐ray spectroscopy (EDS) analysis revealed strong F signals in the deposition. Consistent with the ATR‐FTIR results, these findings confirm that as the depth of overdischarge increases, LiPF_6_ in the electrolyte decomposed and precipitated. Further, transmission electron microscopy (TEM) measurements were conducted to examine Cu ion dissolution and SEI changes. In degraded cells, the graphite anode was coated with a thick SEI layer of approximately 40–50 nm (Figure 3a). After COD treatment, the SEI thickness decreased significantly to 2.9 nm (Figure 3b), indicating partial decomposition of the SEI. In contrast, Deep OD further reduced the SEI to 2.2 nm and yielded a non‐uniform layer (Figure 3c). Cu was detected in the graphite after Deep OD (Figure S12), indicating that Cu species dissolved and redeposited. Furthermore, the resulting Li^+^ can cause structural damage through excessive insertion into cathode. Such reactions could impair subsequent battery cycling performance.

In situ Raman spectroscopy was used to monitor SEI evolution on the graphite surface. The disorder of the graphite anode increased with the depth of the overdischarge (Figure 3d; Figure S13). This behavior is attributed to SEI decomposition and the accumulation of Cu dissolution and deposition during overdischarge, rather than intrinsic defects within the graphite itself [38]. However, the D band intensity slightly decreased again when close to Deep OD, likely because a large fraction of the SEI had already decomposed and additional side reactions had occurred. Accordingly, I_D_/I_G_ ratio progressively increased from 0.194 to 0.369 with overdischarge depth, peaking at 0.01 V. These findings reflected a gradual weakening of the G band and strengthening of the D band due to the SEI decomposition and increased disorder. Nevertheless, contribution from Cu ion dissolution may also have been involved under Deep OD conditions. The slight decrease in the I_D_/I_G_ ratio at Deep OD, 0.362, supported the impedance results, suggesting the possibility of Cu dissolution and deposition. ICP‐MS tests were conducted on the graphite anode (Figure 3e). The results indicate that increasing the depth of overdischarge extract Li^+^ from the SEI and may lead to side reactions together with excessive decomposition of SEI.

X‐ray photoelectron spectroscopy (XPS) analysis was performed to further probe the SEI evolution during overdischarge. As shown in Figure S14a (Supporting Information), the Li 1s spectrum confirmed that the SEI layer was present on the graphite surface under all conditions. As shown in Figure 3f and Figure S14b (Supporting Information), characteristic C─C (284.6 eV) and C─H (285.2 eV) peaks were observed in the C 1s spectrum. For the degraded anode, strong peaks corresponding to C─O (286.7 eV) and O─C═O (288.9 eV) were observed, which can be attributed to ether or carbonate groups such as lithium alkyl carbonates (ROCO_2_Li) or polycarbonates (ROCO_2_R’) [39, 40]. A peak at higher binding energy corresponds to the existence of SEI [39, 40, 41]. After COD, the C─O and O─C═O peaks weakened, confirming partial decomposition of organic lithium species. In contrast, Deep OD resulted in a resurgence of these peaks, caused by electrolyte decomposition and reformation of organic SEI components. This result was supported by the O 1s spectrum (Figure 3g; Figure S14c). In the degraded graphite anode, peaks corresponding to Li─O (530.6 eV) and Li_2_CO_3_ (531.6 eV) appeared at lower binding energy [41], whereas peaks at 532.7 and 533.5 eV are assigned to C─O and ROCO_2_Li, respectively, indicating the formation of a mixed organic and inorganic SEI on the degraded graphite surface [39, 40]. Under COD, the weakened ROCO_2_Li peak supports that overdischarge can decompose the SEI. Deep OD, however, showed stronger ROCO_2_Li peaks and decreased C═O signals, suggesting exposure of inorganic species and additional organic compound formation, likely from electrolyte decomposition. These organic‐related peaks are considered to originate from carbonate solvent decomposition, generating fresh ROCO_2_Li and polycarbonate species distinct from the pristine SEI, which may subsequently alter Li^+^ kinetics. Analysis of the F 1s spectrum (Figure S14d) further revealed that the presence of LiF (684.7 eV) and Li_x_PO_y_F_z_ (686.1 eV) in the degraded graphite anode confirmed the mixed nature of the SEI [42, 43, 44]. Additional C─F and Li_x_PF_y_ (687.9 eV) peaks appeared under Deep OD, implying further SEI decomposition and either binder exposure or electrolyte decomposition [45]. No Cu signals were detected on the XPS probed surface (Figure S14e). Collectively, these results demonstrate that COD facilitates selective decomposition of the SEI layer while mitigating electrode damage, Cu dissolution and extensive electrolyte decomposition (Figure 3h). Nevertheless, since overdischarge may exert distinct effects on the cathode, its evaluation remains essential.

**FIGURE 4:**
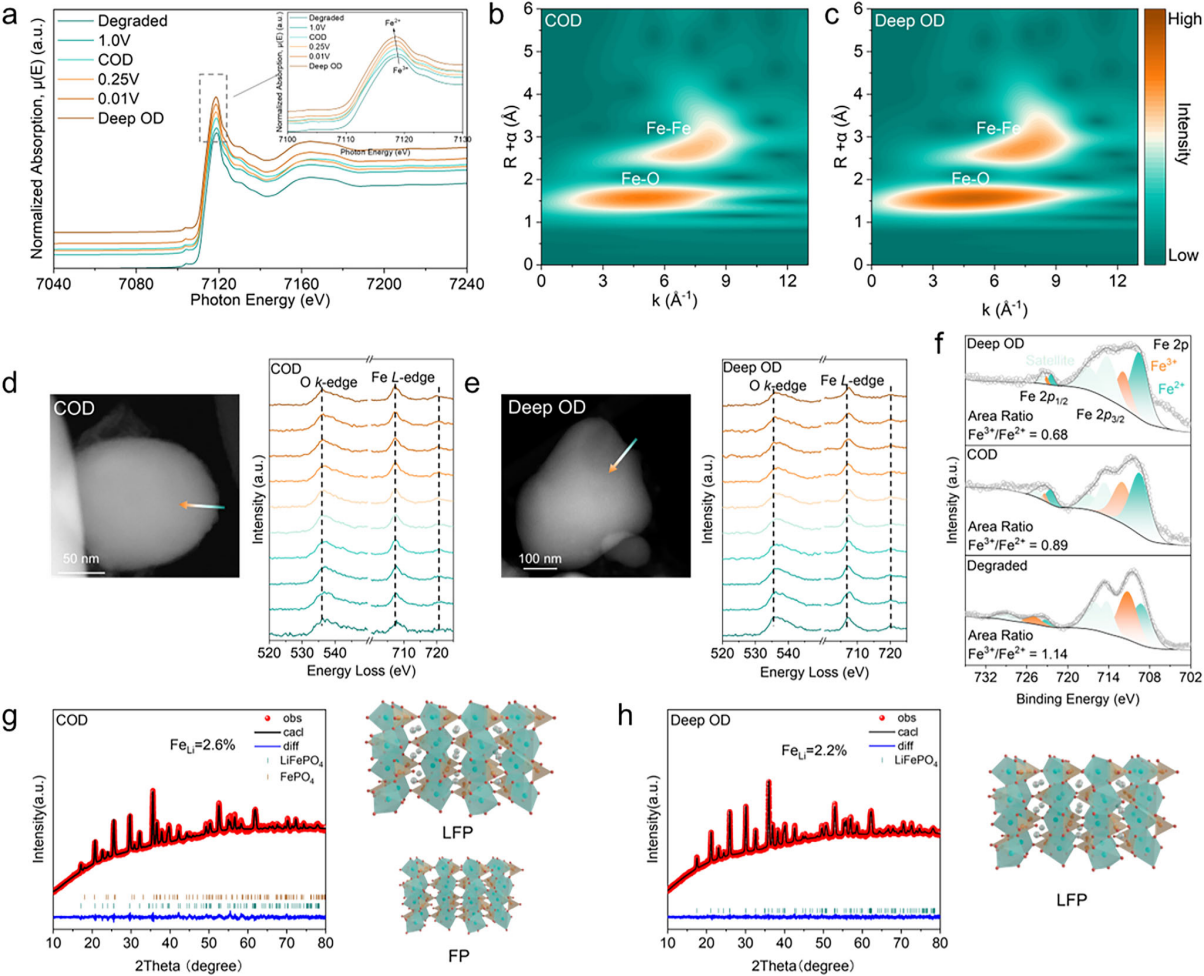
Structural and chemical evolution of LFP under different overdischarge conditions. (a) The normalized Fe K‐edge XANES spectrum of different conditions LFP samples and (b,c) wavelet transform of the EXAFS spectra of COD and Deep OD LFP; (d,e) HAADF‐STEM images of COD and Deep OD LFP, with corresponding EELS line scan results across the interface; (f) Fe 2p XPS spectra of Degraded, COD and Deep OD LFP; (g,h) Rietveld refinements from ex situ XRD patterns of COD and Deep OD LFP.

To understand the changes on the cathode side such as CEI evolution and particle integrity, SEM‐EDS analysis of the cathode was conducted. The results revealed no significant morphological changes or Cu signals (Figure S15), although such analysis may overlook electronic or structural modifications. To probe these changes, the Fe K‐edge XANES spectra exhibited a slight shift toward lower energy with increasing overdischarge depth (Figure 4a), demonstrating partial reduction of Fe^3+^ to Fe^2+^ in the LFP structure [46]. This observation confirmed that Li^+^ released from SEI decomposition during overdischarge is partly reinserted into the cathode and facilitates partial Fe^3+^ reduction. Wavelet‐transformed of the EXAFS spectra further revealed that the reduction of Fe^3+^ to Fe^2+^ increased structural symmetry while modifying local bonding distances and disorder (Figure 4b,c; Figure S16) [47].

To investigate the microstructural effects, TEM imaging was performed on Degraded, COD‐treated, and Deep OD LFP cathodes. Both the Degraded and COD samples retained a stable morphology and an intact CEI (Figure S17a,b). In contrast, Deep OD induced particle cracking (Figure S17c), likely arising from the excessive Li^+^ insertion, supporting that COD effectively decomposes SEI without damaging the cathode. Electron energy loss spectroscopy (EELS) was performed to probe changes in Fe valence state and elemental distributions near the particle surface. In Degraded LFP, shifts in the Fe L_2_ and L_3_ edges are strong indicators of Fe valence changes, while O K‐edge reflects electronic transitions from O 1s to unoccupied O 2p orbitals [9]. In the Degraded LFP (Figure S18), the O K‐edge and Fe L‐edges were unstable across the scan line. After COD (Figure 4d), the Fe L‐edges shifted, suggesting partial reduction of Fe^3+^ to Fe^2+^ via Li^+^ insertion. Under Deep OD (Figure 4e), further reduction and stabilization of spectra were observed, confirming a higher degree of Fe reduction. XPS was carried out to determine the surface valence states of Fe in LFP. The relative areas of the Fe 2p_3/2_ peaks corresponding to Fe^2+^ and Fe^3+^ were used to quantify phase composition [9, 12]. exhibited FePO_4_ (FP) phase, with area ratios of 1.14 (degraded) and 1.01 (1.0 V). The ratio of COD is reduced to 0.89, indicating significant restoration of the LFP phase. Continuing, the ratio decreased as the depth of overdischarge increased to 0.87 (0.25 V), 0.71 (0.01 V), and 0.68 (Deep OD) (Figure 4f; Figure S19a), indicating more extensive Li^+^ insertion and structural recovery as overdischarge depth increased. Although ICP‐MS detected trace amounts of Cu ions, no Cu signal was observed in XPS due to the surface sensitive nature of the technique (Figure S19b).

Interestingly, X‐ray diffraction (XRD) revealed that the increasing depth of overdischarge transformed the S‐LFP cathode into a pure LFP phase without residual FP or impurity phases (Figure S20a), indicating that overdischarge reduces Li/Fe antisite defects in S‐LFP. As the above discussion on graphite anode did not include XRD analysis, which may raise concerns regarding the possibility that COD protocol negatively impacts its structural integrity. To address this concern, we also performed XRD measurements on the graphite anode under the same overdischarge conditions as applied to the LFP cathode. The results confirmed that overdischarge induces no structural damage on the graphite anode (Figure S20b). These findings demonstrate that the COD protocol can selectively repair structural defects in the LFP cathode through lithium reinserted while maintaining the stability of the graphite anode. Rietveld refinement was performed to quantify crystalline order and antisite defects. The degraded cathode contained 3.3% antisite defects (Figure S21a and Table S3), which slightly decreased to 3.2% after 1.0 V discharge (Figure S21b and Table S4). Notably, COD further reduced defects to 2.6% (Figure 4g; Table S5), and with increasing overdischarge depth to 2.4% at 0.25 V and 2.2% at 0.01 V (Figure S21c,d and Tables S6 and S7). However, no further reduction was observed in Deep OD (Figure 4h; Table S8); this is closely related to the limitation in the amount of extractable Li^+^ within the SEI and side reactions induced by overdischarge. While a higher overdischarge voltage intensifies SEI decomposition, it potentially triggers severe side reactions. It might hinder the pathways for Li^+^ reinsertion into the LFP cathode, which is led by Cu dissolution and redeposition. Consequently, these detrimental effects appear to have prevented further recovery of structural defects by overdischarge.

Combined analysis of the graphite and LFP electrodes highlighted the dual role of overdischarge in rejuvenating S‐LFP. COD selectively decomposes the organic components of the SEI and releases Li^+^ from the graphite anode, which is subsequently reinserted into the cathode and heals structural defects without damaging particles, decomposing the CEI, dissolving Cu or decomposing electrolyte. By contrast, Deep OD causes electrolyte decomposition, particle cracking, and Cu dissolution.

### 2.4 Techno‐Economic and Environmental Assessment

**FIGURE 5:**
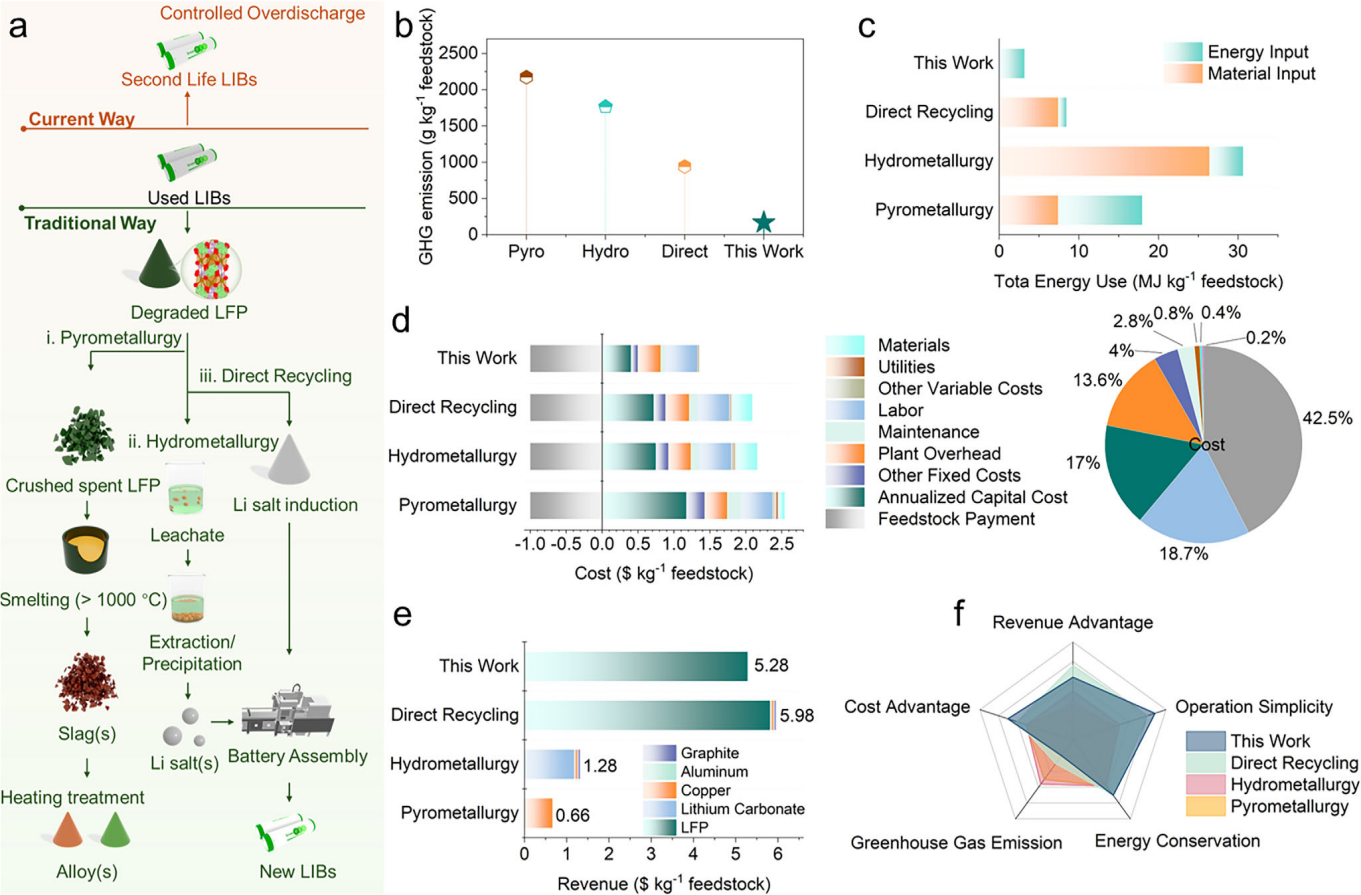
Techno‐economic analysis of different LIB recycling technologies. (a) Schematic illustration of the traditional metallurgy‐based recycling methods, direct recycling and current proposed recycling methods; (b) GHG emission and (c) total energy requirements for recycling; (d) Techno‐economic analysis results of pyrometallurgy, hydrometallurgy, direct recycling and current recycling methods and (e) revenue analysis; (f) Comprehensive comparison of different battery recycling technologies.

To evaluate the industrial viability of the COD method, techno‐economic and environmental assessments were carried out against conventional metallurgical and direct recycling routes [7, 48, 49, 50, 51]. As illustrated in Figure 5a, a simplified flowchart outlines the key recycling methods. Conventional pyrometallurgy and hydrometallurgy recover high‐value transition metals such as Ni, Co, and Li, but for LFP the low intrinsic value of Fe and P renders these approaches both economically unattractive and environmentally intensive due to their high energy and chemical consumption. Direct recycling partially alleviates these issues by preserving the olivine structure and requiring only a relithiation step, yet the process still depends on battery disassembly and tight impurity control, making large‐scale implementation challenging. By contrast, COD achieves rejuvenation without disassembly, simplifying process flows and avoiding black mass control.

As shown in Figure 5b, this translates into substantially lower greenhouse gas (GHG) emissions and energy consumption compared with pyrometallurgy and hydrometallurgy, while maintaining comparable performance. Additionally, COD provides a direct pathway to rejuvenate S‐LFP, which minimizes secondary waste and reduces costs associated with recycling. These combined advantages highlight COD as a uniquely scalable and sustainable strategy for the specific resource profile of S‐LFP, positioning it as a viable alternative to conventional recycling methods. Simulations using Argonne National Laboratory's Everbatt 2023 model compared the impact of treating 10 000 tons of S‐LFP battery cells across distinct recycling pathways, each with its own process chemistry and reagent consumption, under US market conditions (Tables S9 and S10). All values for GHG emissions, energy consumption, and costs are normalized per kilogram of feedstock.

In terms of GHG emission (Figure 5b; Table S11), pyrometallurgical recycling emitted approximately 2173 g kg^−1^ GHG significantly more than the 1777 g kg^−1^ associated with hydrometallurgy. Direct recycling emitted about 941 g kg^−1^, lower than metallurgical methods, owing to reduced material consumption and simplified operating procedures. By contrast, the proposed COD method, which relies solely on electricity, produced merely 168 g kg^−1^ of GHG emissions, making it the environmentally friendly pathway.

Energy consumption (Figure 5c; Table S11) further underscored our proposed method. Pyrometallurgy required approximately 15.7 MJ kg^−1^ energy use, largely for high‐temperature processes, while hydrometallurgy consumed 23.3 MJ kg^−1^ due to energy‐intensive liquid handling. Whereas direct recycling required roughly 9.0 MJ kg^−1^, COD was by far the most energy‐efficient, consuming 3 MJ kg^−1^. The electricity consumption for the COD protocol was estimated by experimentally measuring the electrical energy consumed during the COD protocol using a battery tester. Additionally, to account for the uncertainty in the S‐LFP during the actual collection process, a conservative assumption was made that the COD protocol would begin with the battery in a fully charged state.

Based on these inputs, recycling cost and corresponding revenue were modeled (Figure 5d). Pyrometallurgy required approximately $2.5 kg^−1^ in processing costs, with a large portion attributed to the annualized capital costs. Hydrometallurgy follows closely at $2.3 kg^−1^. Direct recycling requires around $2.1 kg^−1^, while COD requires roughly only $1.3 kg^−1^, nearly half the cost of conventional methods. Labor constitutes the largest cost proportion in the COD approach, mainly for manual operating the overdischarge procedure on degraded batteries. As shown in Figure 5e, Pyrometallurgy yielded only about $0.66 kg^−1^ in revenue, mainly from Cu recovery from the anode current collector. Hydrometallurgy performed slightly better, generating about $1.28 kg^−1^, primarily due to lithium recovery as lithium carbonate. However, direct recycling was much more profitable, generating approximately $5.98 kg^−1^ by directly recovering usable LFP materials without metal reprocessing. COD generated $5.28 kg^−1^, slightly lower but still significantly exceeding traditional metallurgical approaches. For a conservative revenue assessment, we assumed that the battery was disassembled after COD and excluded graphite and Cu current collectors, which are prone to contamination by COD. Furthermore, we also excluded aluminum current collectors to maintain rigorous evaluation. Since we focused on the rejuvenation of LFP batteries, revenues were estimated based on the LFP materials only. Specifically, we assumed that materials degraded to 80% of their original capacity could be rejuvenated to approximately 90% through COD, compared to a 100% capacity recovery typically achieved by direct recycling.

Despite the slightly lower profitability, COD combines the lowest cost, lowest GHG emissions, and the highest energy efficiency, making it a commercially viable and sustainable strategy for LFP recycling (Figure 5f). Both direct recycling and COD deliver revenues of at least $5 kg^−1^, representing nearly three times the returns of pyrometallurgical or hydrometallurgical routes, thereby positioning COD as a practical and scalable alternative.

## 3 Conclusion

This work demonstrates that controlled overdischarge (COD) provides an effective and non‐invasive strategy to rejuvenate S‐LFP. By selectively releasing Li^+^ from the SEI, COD reduces Li/Fe antisite defects and mitigates critical reactions. As a result, this approach achieves 9.56% capacity recovery and extends the lifespan by 214 cycles, delivering markedly improved techno‐economic impacts compared to current recycling pathways. The protocol can be repeated without structural compromise, reframing overdischarge as a practical tool for next‐generation lithium‐ion battery recycling.

## Conflicts of Interest

The authors declare no conflicts of interest.

## Supporting information




**Supporting File**: adma72431‐sup‐0001‐SuppMat.docx.

## Data Availability

The data the support the findings of this study are available from the corresponding author upon reasonable request.
