# Supplementary material for: Efficient Non‐Invasive Rejuvenation of Spent Lithium Iron Phosphate Batteries Through Controlled Overdischarge
Source: Adv Mater. 2026 Feb 10;38(15):e22927. doi: 10.1002/adma.202522927 (PMC12983439; doi:10.1002/adma.202522927)
Supplement: Supplementary file 1 — Supporting File: adma72431‐sup‐0001‐SuppMat.docx. [file ADMA-38-e22927-s001.docx]

**Supporting Information**

for

Efficient Non-Invasive Rejuvenation of Spent Lithium Iron Phosphate Batteries Through Controlled Overdischarge

Jinu Song, Yujie Chen, Nianji Zhang, Cancan Peng, Huan Li, Chao Ye*, and Shi-Zhang Qiao*

J. Song, Y. Chen, N. Zhang, C. Peng, H. Li, C. Ye, and Prof. S.-Z. Qiao

School of Chemical Engineering, The University of Adelaide, Adelaide, SA 5005, Australia

E-mail: [chao.ye@adelaide.edu.au](mailto:chao.ye@adelaide.edu.au); [s.qiao@adelaide.edu.au](mailto:s.qiao@adelaide.edu.au)

1. **Experimental Section**

**Chemical and materials**

All chemicals were used as received without additional purification. The electrolyte solution was sourced from Sigma-Aldrich. Commercial cylindrical batteries are purchased from Jaycar Electronics, Australia.

**Collection of degraded battery**

Spent lithium iron phosphate (LFP) and graphite electrode were collected from cylindrical 18650 Li-ion batteries (Lithium Iron Phosphate vs Graphite, 1600 mAh 3.2 V) which are cycled following specification between 2.5–3.65 V with 1600 mA (Constant Current mode) until it retains 80% of the initial capacity.

**Overdischarge enabled rejuvenation on coin cell**

Spent LFP batteries disassembled in the Ar-filled glove box for coin cell test with O_2_ and H_2_O levels controlled below 0.1 ppm. Double sided coated electrode sheet was carefully scraped on one side to be used in coin cell. Electrodes were punched into circular discs with diameters of 10 mm for LFP cathode and 12 mm for graphite anode. CR2032 type coin cells were assembled within the same glove box. Celgard 2340 membrane (Ø 19 mm) was used as the separator, and 50 µL of electrolyte consisting of 1.0 M LiPF_6_ dissolved in a 1:1 vol. ratio of EC/DEC was used. The electrochemical testing of the coin cell is performed using Neware battery testers at 28°C. Galvanostatic charge-discharge (GCD) tests were conducted at 0.3 C for the first five cycles. Subsequently, cells were slowly overdischarged to 0.5 V (Constant Current and Constant Voltage mode). Afterward, a rest period allowed internal lithium-ion concentration equilibrium and polarization self-healing. A few additional slow charge/discharge cycles were performed to refine the SEI layer before regular cycling. Long term performance was finally evaluated by cycling at 0.3 C within the 2.5-4.0 V range.

**Overdischarge enabled rejuvenation on cylindrical cell**

Commercial cylindrical cell was used after being degraded by performing charge/discharge according to the above conditions by LAND battery testers at 28°C. The same overdischarge protocol used for coin cells was applied here, involving discharge to 0.5 V (Constant Current and Constant Voltage mode), followed by a rest period to allow internal self-healing, and then several low-rate cycles to reconstruct a robust solid electrolyte interphase (SEI). Long term performance was evaluated by cycling at 1 C within the 2.5-3.65 V range (Constant Current mode).

**Electrochemical measurements**

Electrochemical impedance spectroscopy (EIS) measurements were performed using a Biologic VMP-3e potentiostat within a frequency range of 1 MHz to 10 mHz, employing a 5 mV amplitude perturbation. Cyclic voltammetry (CV) was conducted at various scan rates within a voltage range of 2.5–4.0 V on the same potentiostat. It was carried out with scan rates ranging from 0.1 mV s^-1^ to 1 mV s^-1^. The lithium diffusion coefficient was determined using the Randles-Sevcik equation.

$$i_{p}=2.69\times{10}^{5}{An}^{3/2}C_{0}^{1/2}D^{1/2}v^{1/2}$$

where i_p_ represents the peak current (A), A denotes the electrode surface area (cm^2^), n refers to the number of electrons transferred, C_0_ is the lithium-ion concentration during the reaction process (mol cm^-3^), D represents the diffusion coefficient (cm^2^ s^-1^), and v is the scan rate in the CV tests (V s^-1^).

Galvanostatic intermittent titration technique (GITT) was conducted on Neware battery tester with chare-discharge using 0.1 mA for 5 min each, followed by a 2 min relaxation. GITT calculated by following equation,

$$D_{\mathrm{Li}}=\frac{4}{\pi\tau}\left( \frac{m_{B}V_{M}}{M_{B}S} \right)^{2}\left( \frac{\Delta E_{s}}{\Delta E_{t}} \right)^{2}(\tau\ll\frac{L^{2}}{D_{K^{+}}})$$

where τ is the current pulse time, m_B_ is the active material mass, V_M_ is the molar volume of active material, M_B_ is the molecular mass, S is the area of the electrode, ∆E_s_ is the variation of steady state voltage of the cell after a single step, ∆E_t_ is the total transient voltage change of the cell after employing a galvanostatic current I_0_ for the time τ, and L is the average thickness.

**Characterization techniques**

For material characterization, electrode samples were prepared in an Ar-filled glove box and subsequently rinsed with dimethyl carbonate (DMC) to remove surface impurities after coin cell disassembly. X-ray diffraction (XRD) analysis was conducted with a Rigaku Miniflex-600 diffractometer, operating at 40 kV and 15 mA, using Cu-Kα radiation (λ = 0.15418 nm). Attenuated Total Reflectance-Fourier transform infrared spectroscopy (ATR-FTIR) was performed with a Nicolet iS50 (Thermo Fisher) to characterize chemical bonds and functional groups. In-situ Raman tests were conducted with a Renishaw inVia Raman microscope using a 532 nm wavelength laser, recording spectra during slow charge and overdischarge cycling. The electrode microstructure and elemental distributions were characterized using scanning electron microscope (SEM) and energy dispersive X-ray spectroscopy (EDS) mapping performed on a Zeiss Gemini 300. Morphological features of graphite anodes and valence state distributions of LFP cathodes were investigated using scanning transmission electron microscope (STEM, FEI Titan Themis) coupled with electron energy loss spectroscopy (EELS). Synchrotron-based X-ray absorption near-edge structure (XANES) and extended X-ray Absorption Fine Structure (EXAFS) measurements for Fe K-edge and Cu K-edge were conducted in X-ray Absorption Spectroscopy beamline at the Australian Synchrotron. X-ray photoelectron spectroscopy (XPS) characterization was carried out on a K-Alpha XPS system (Thermo Fisher Scientific, USA).

# **Supplemental Figures**


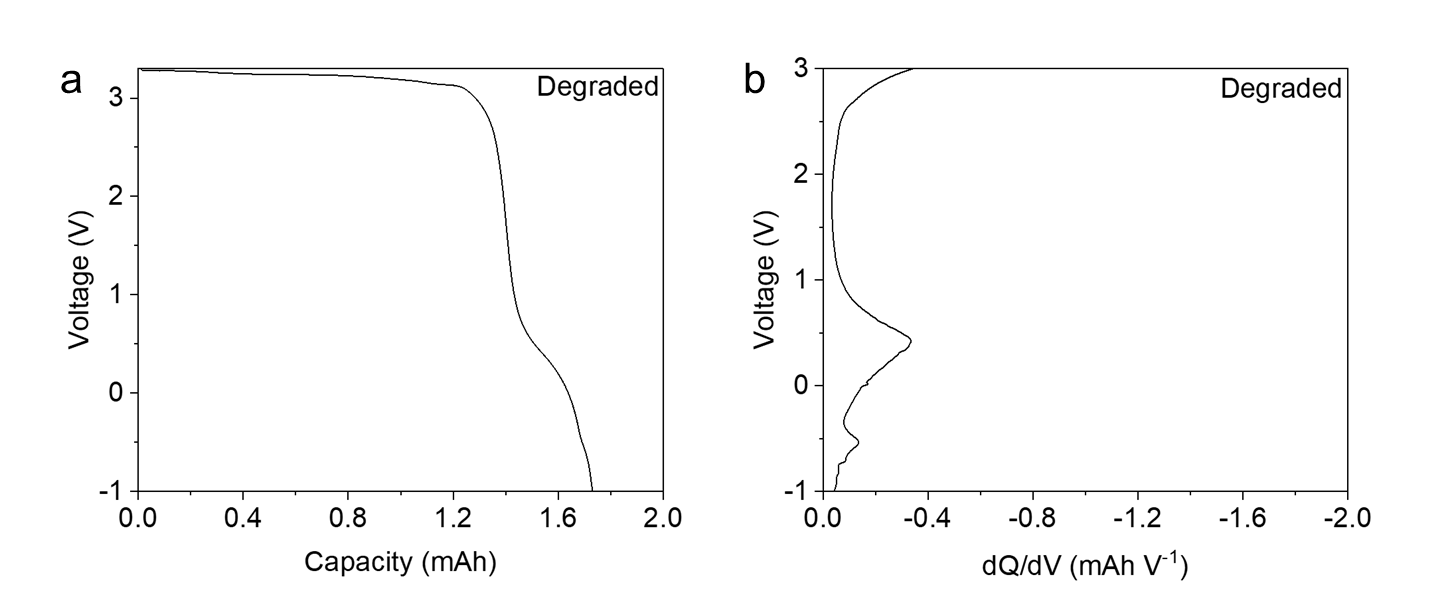


**Figure S1.** a) Galvanostatic discharge curves of cell during overdischarge; b) Corresponding dQ/dV curve of overdischarge cell.


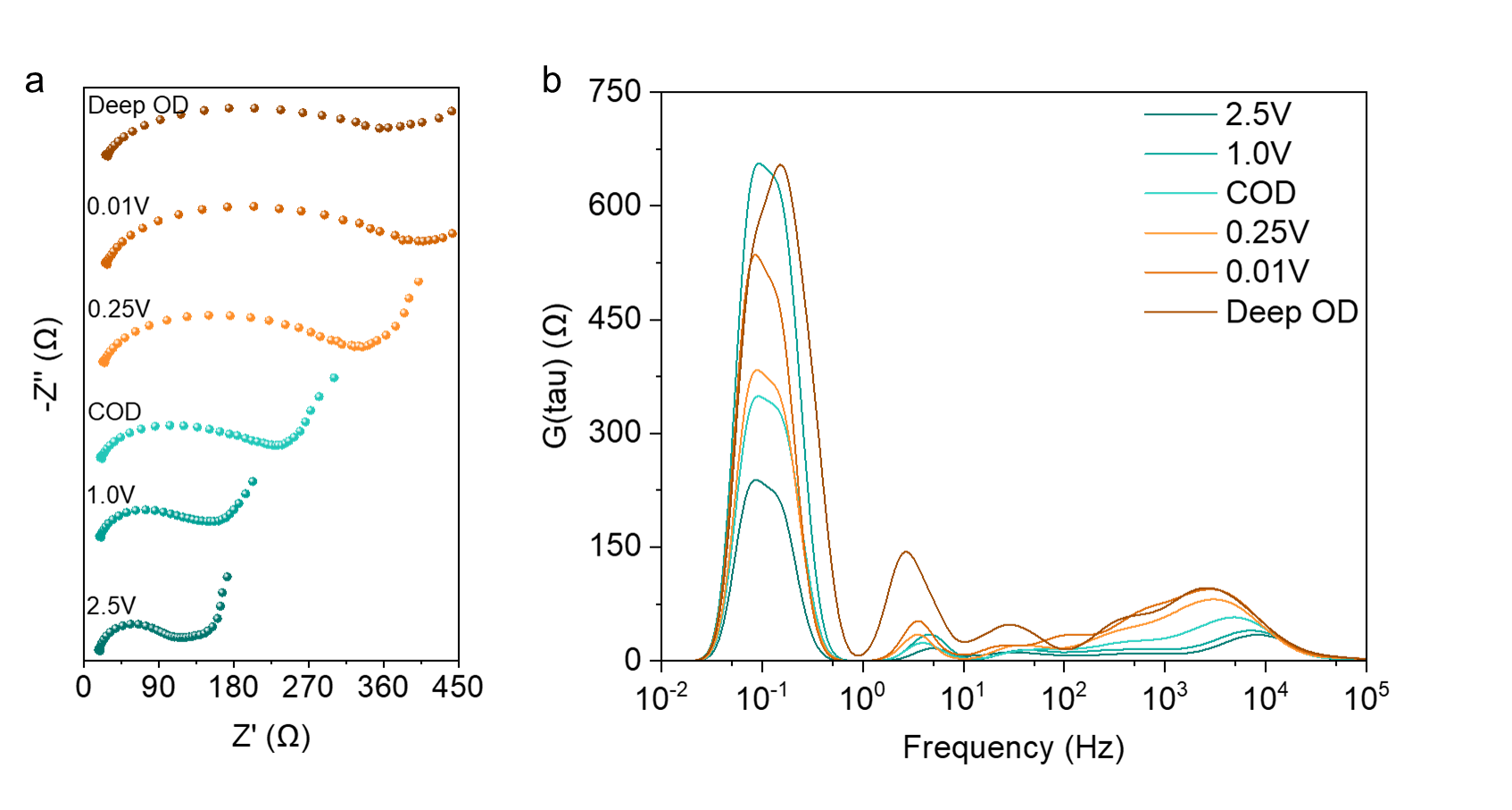


**Figure S2.** a) Nyquist impedance spectra collected during overdischarge; b) Corresponding R_SEI_ value of each discharge voltage; c) Distribution of relaxation times (DRT) for different discharge voltages.


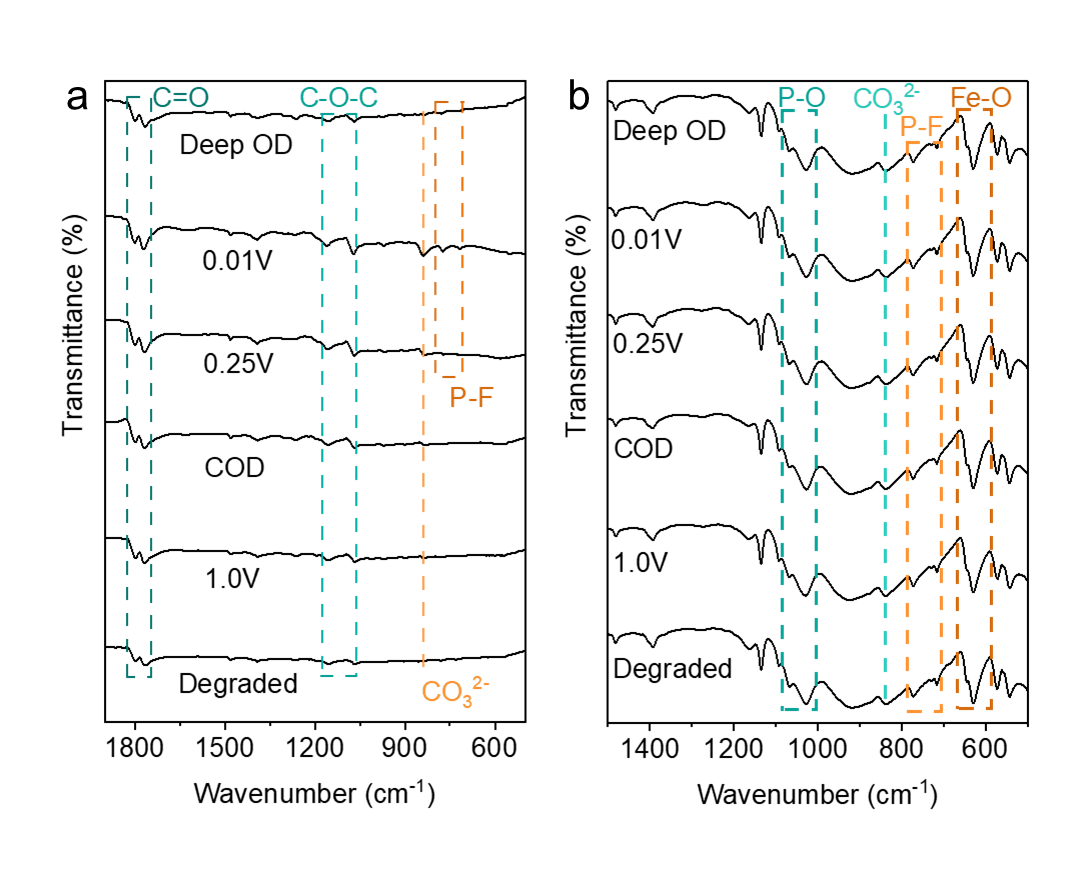


**Figure S3.** FT-IR spectra of a) LFP and b) graphite at different discharge voltages.


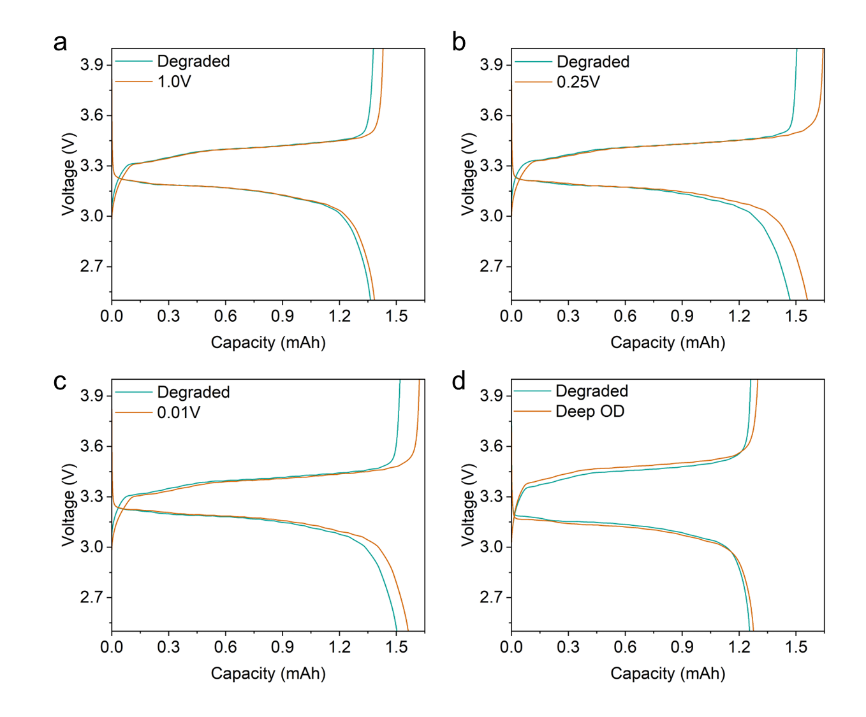


**Figure S4.** GCD curves of a) 1.0 V, b) 0.25 V, c) 0.01 V, and d) Deep OD.


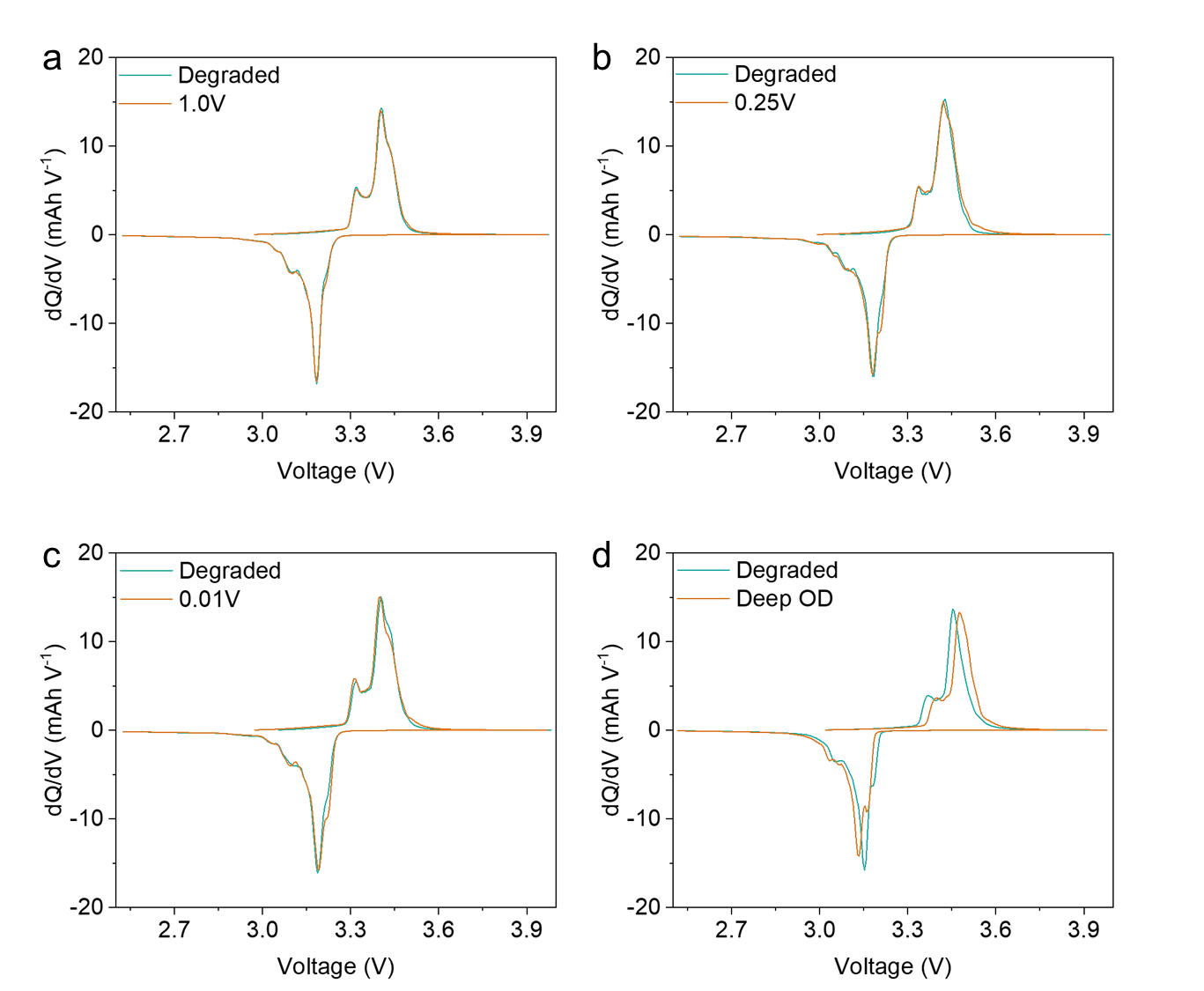


**Figure S5.** dQ/dV profiles of a) 1.0 V, b) 0.25 V, c) 0.01 V, and d) Deep OD.


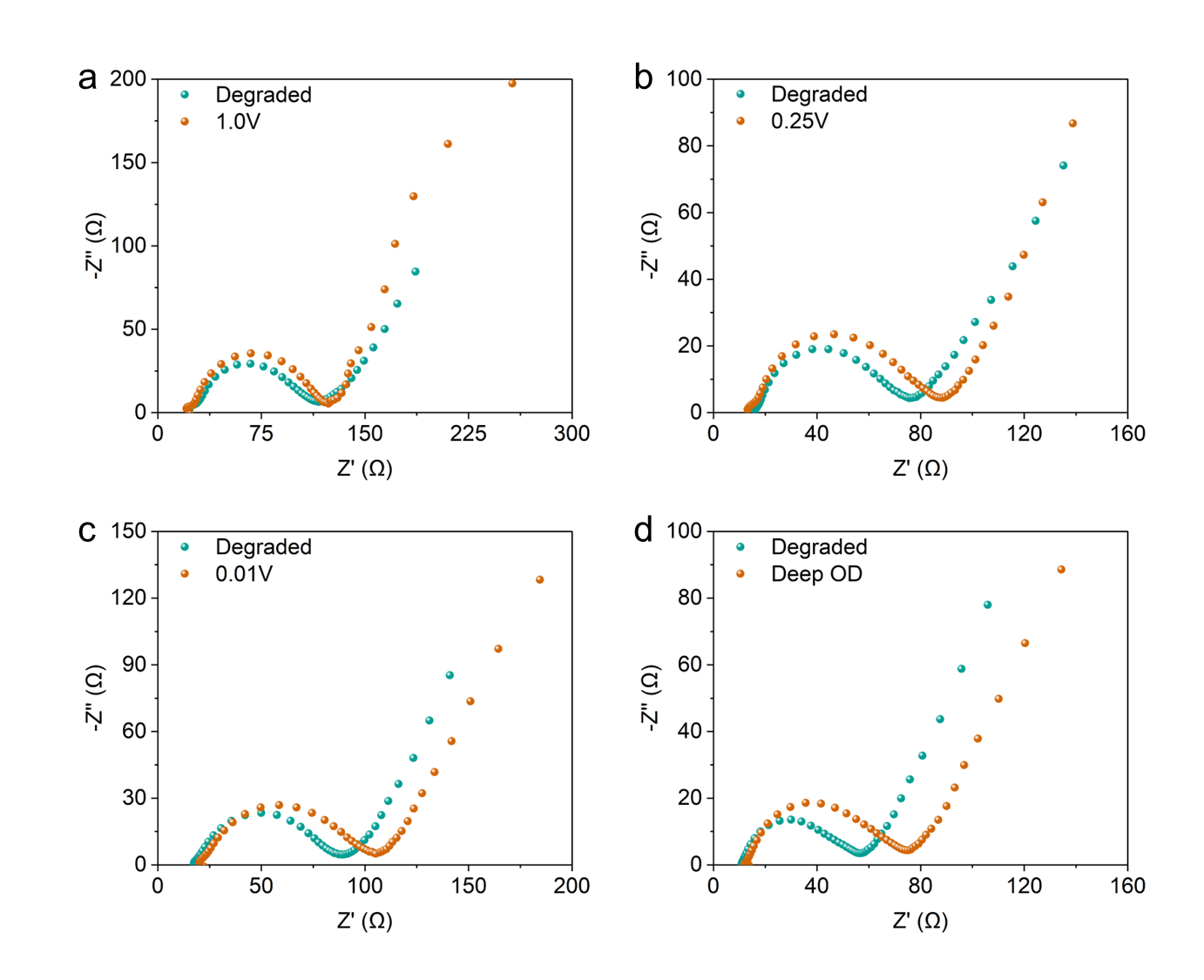


**Figure S6.** EIS of a) 1.0 V, b) 0.25 V, c) 0.01 V, and d) Deep OD.


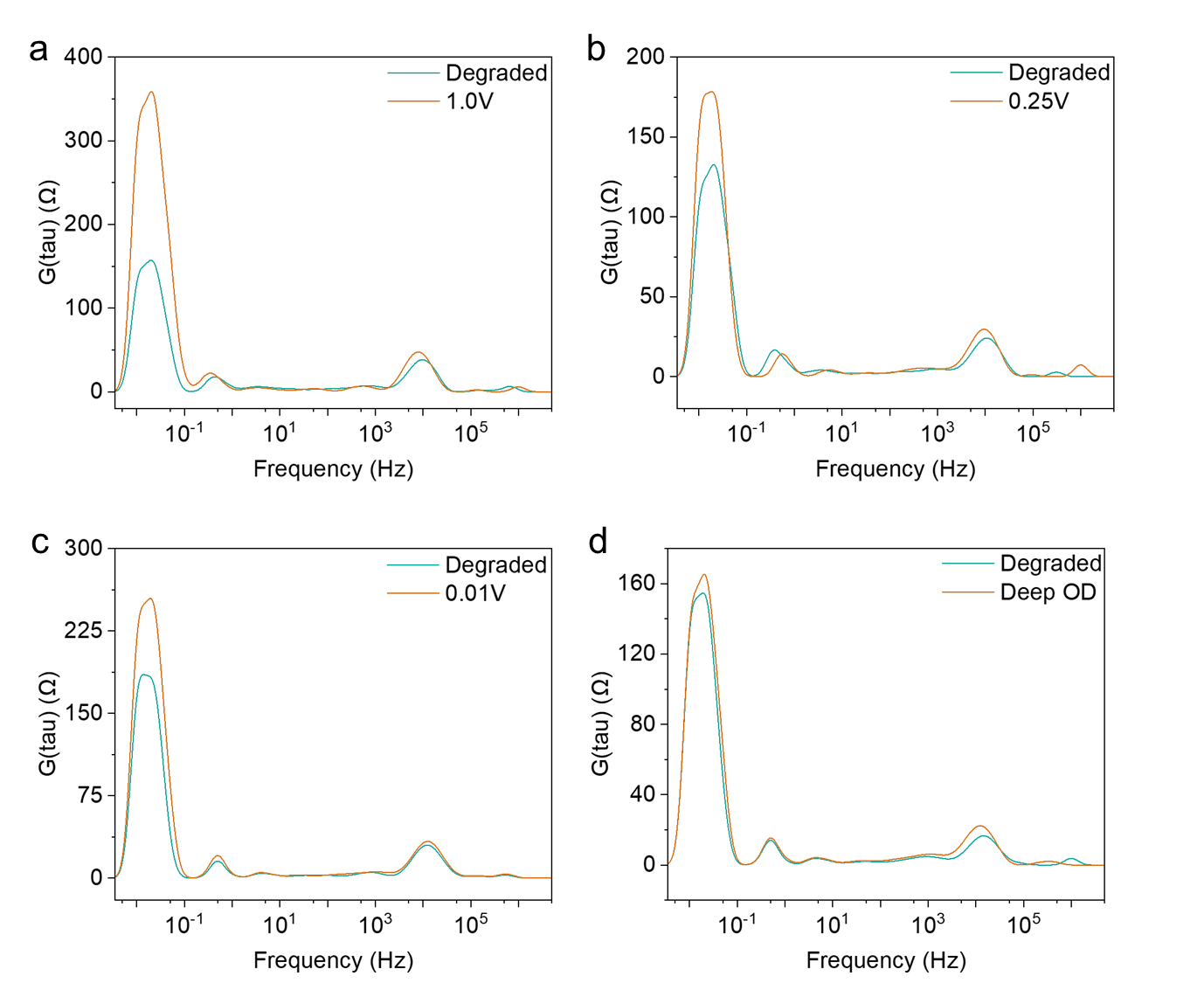


**Figure S7.** DRT spectra of a) 1.0 V, b) 0.25 V, c) 0.01 V, and d) Deep OD.


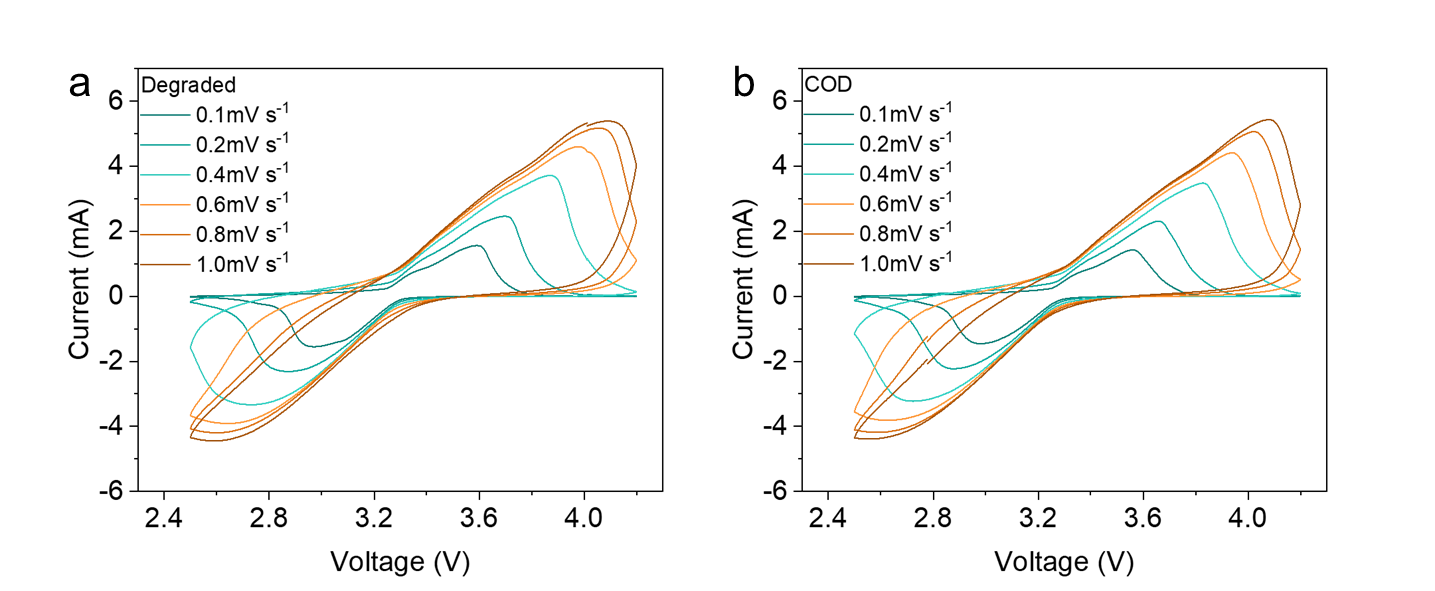


**Figure S8.** CV curves at different scan rates ranging from 0.1 to 1.0 mV s^-1^ a) before and b) after COD.


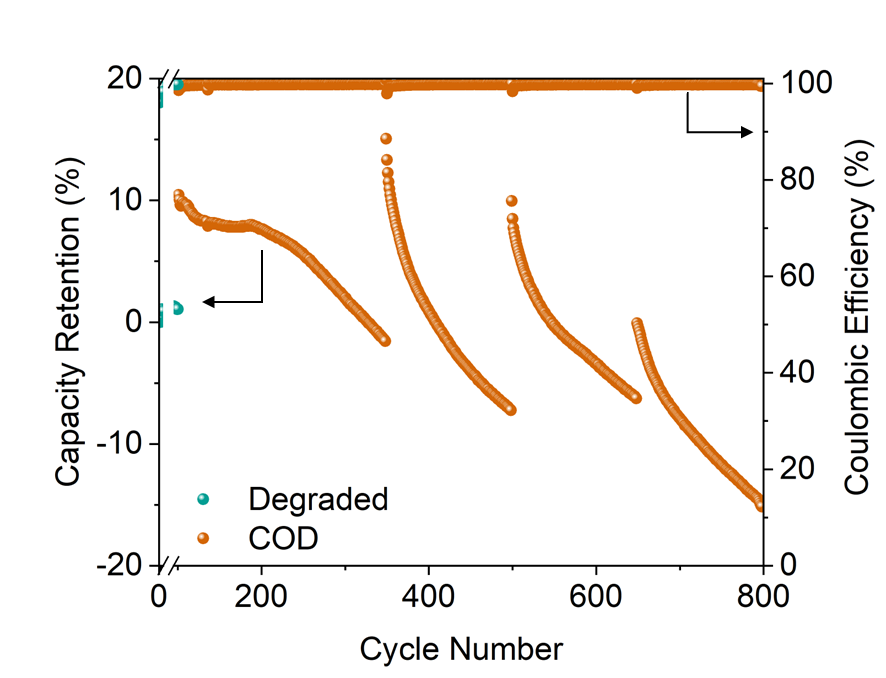


**Figure S9.** Repetitive COD effects on coin cell at 0.3 C.


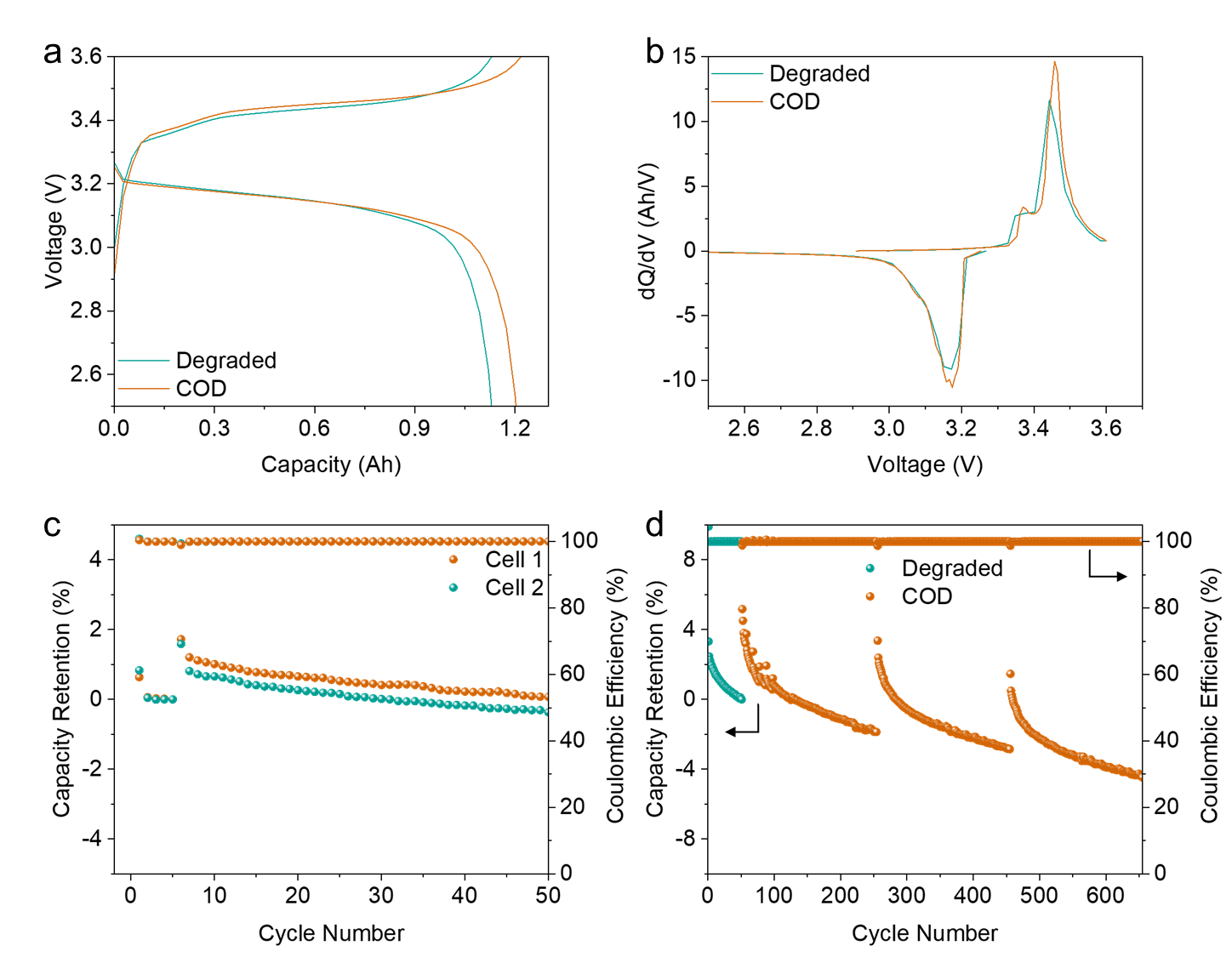


**Figure S10.** Electrochemical performance of overdischarge strategy in 18650 cylindrical battery. a) GCD curves of Cell before and after COD at 1 C; b) Corresponding dQ/dV profiles of before and COD; c) Repetitive COD effects for the same cell; d) Long-term cycling performance of COD at 1 C.


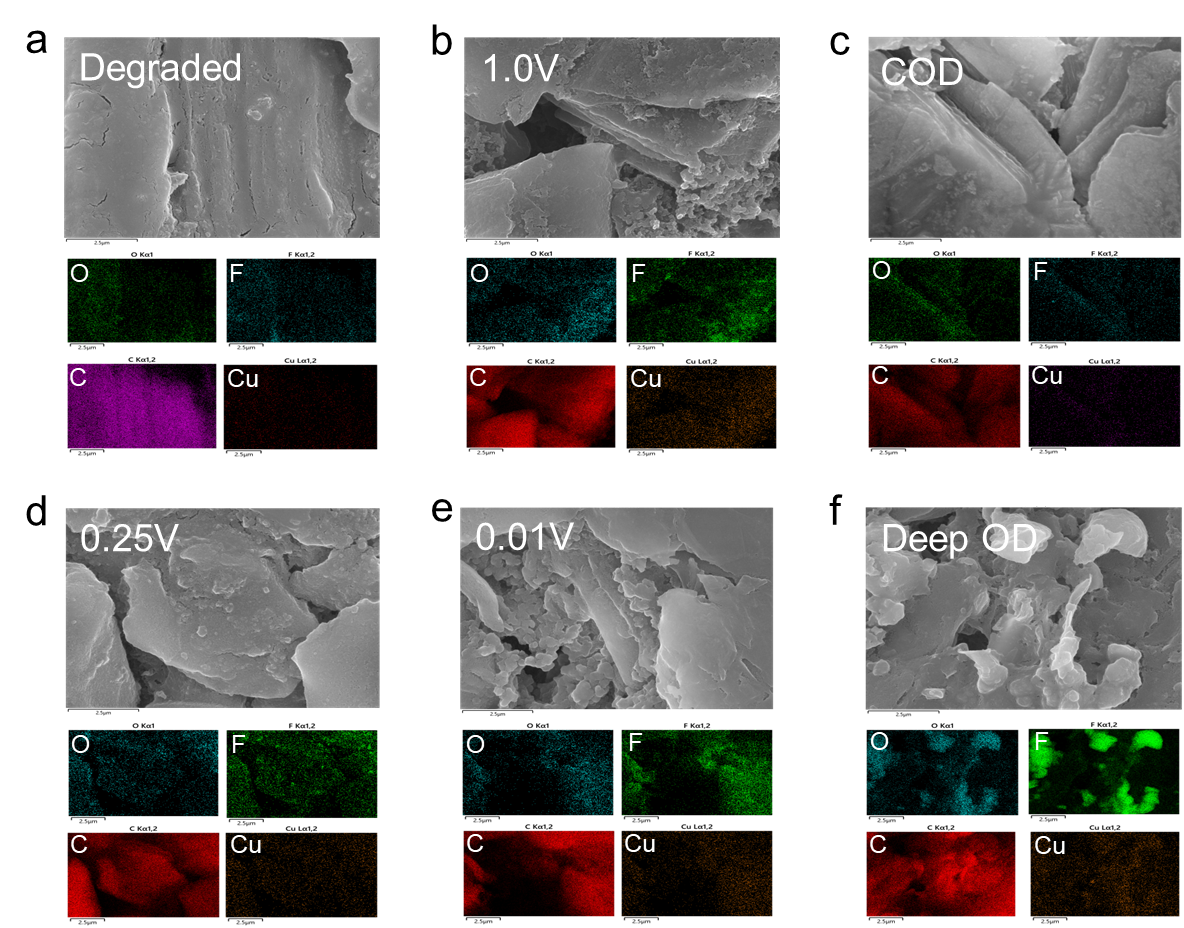


**Figure S11.** SEM image with EDS mapping for graphite in different overdischarge voltage from a) Degraded, b) 1.0 V, c) COD, d) 0.25 V, e) 0.01 V, to f) Deep OD.


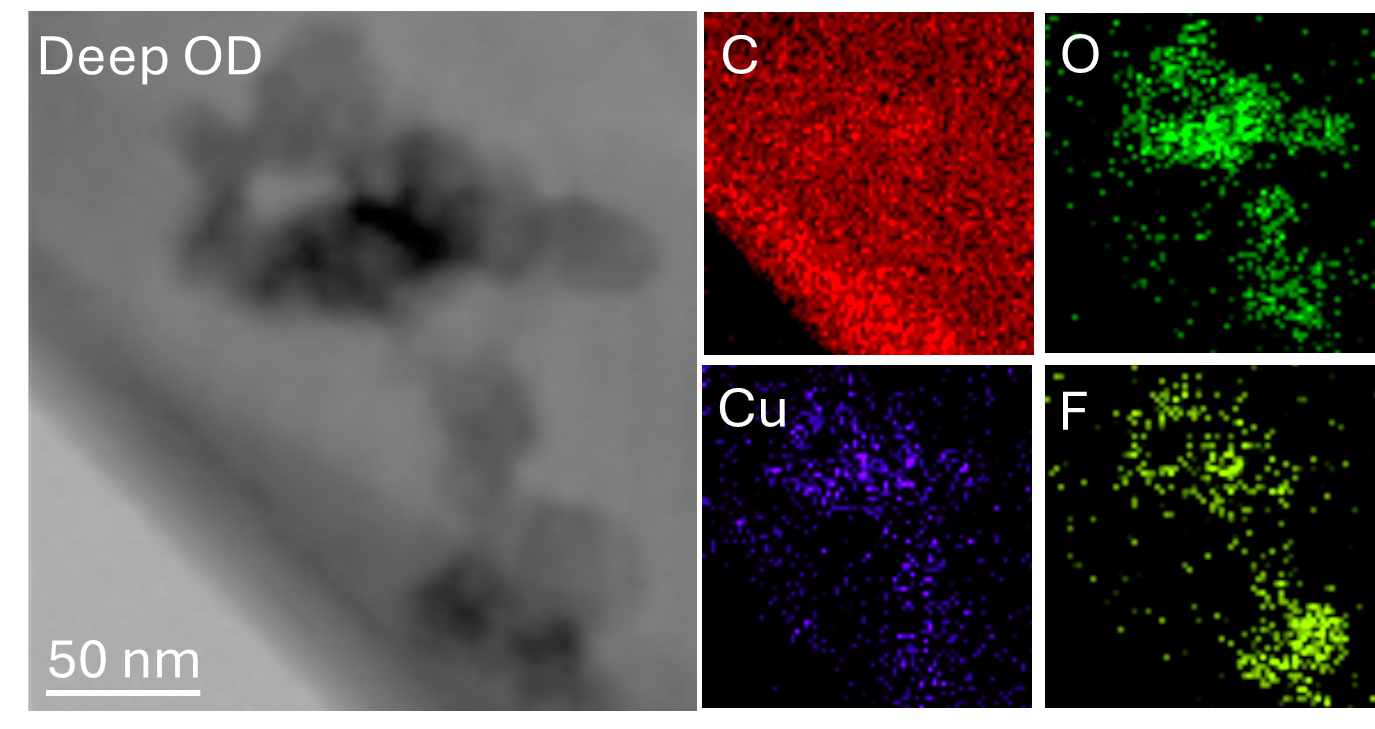


**Figure S12.** TEM image with EDS mapping of Deep OD graphite.


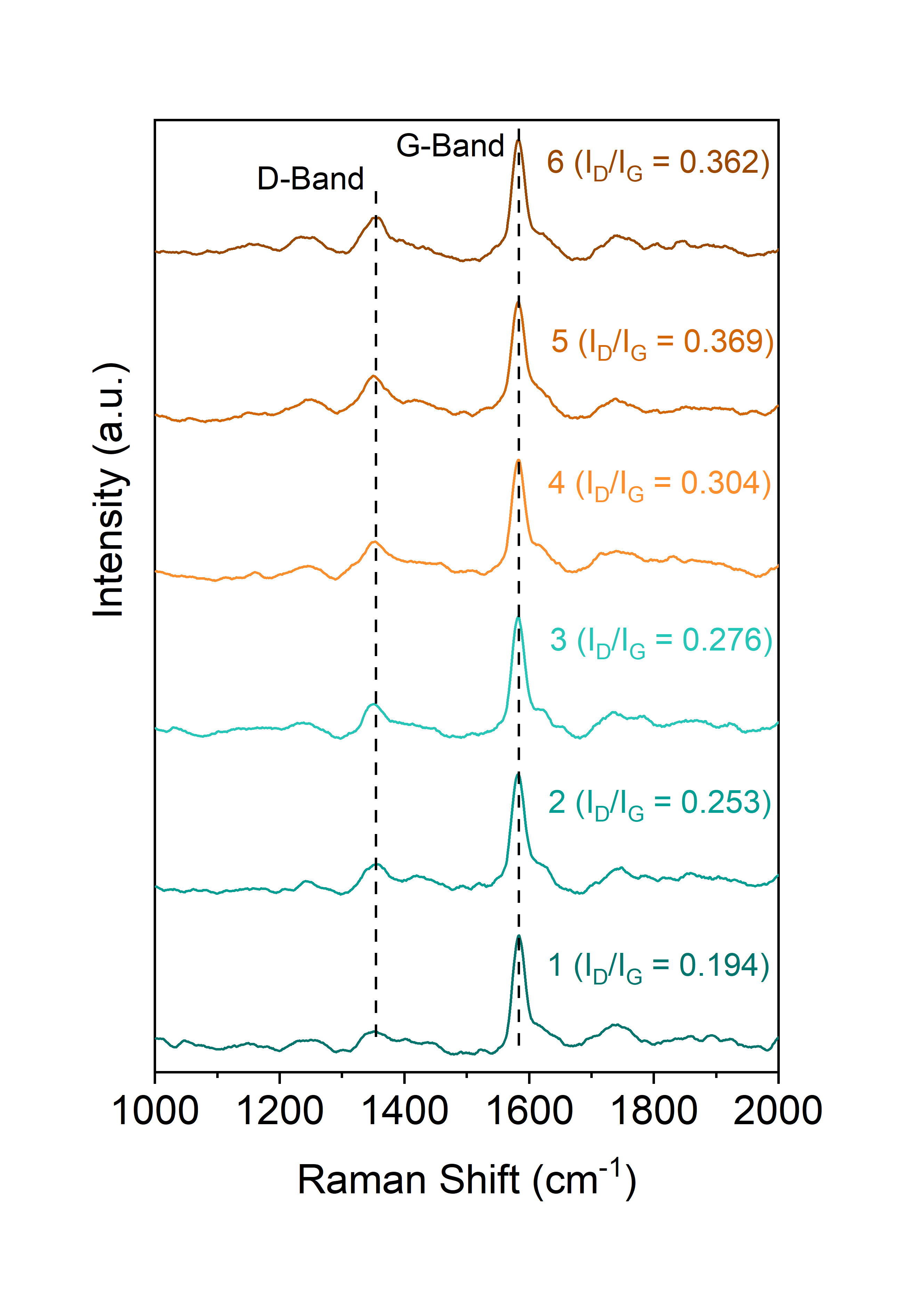


**Figure S13.** The representative Raman I_D_/I_G_ ratio in different spectrum points.


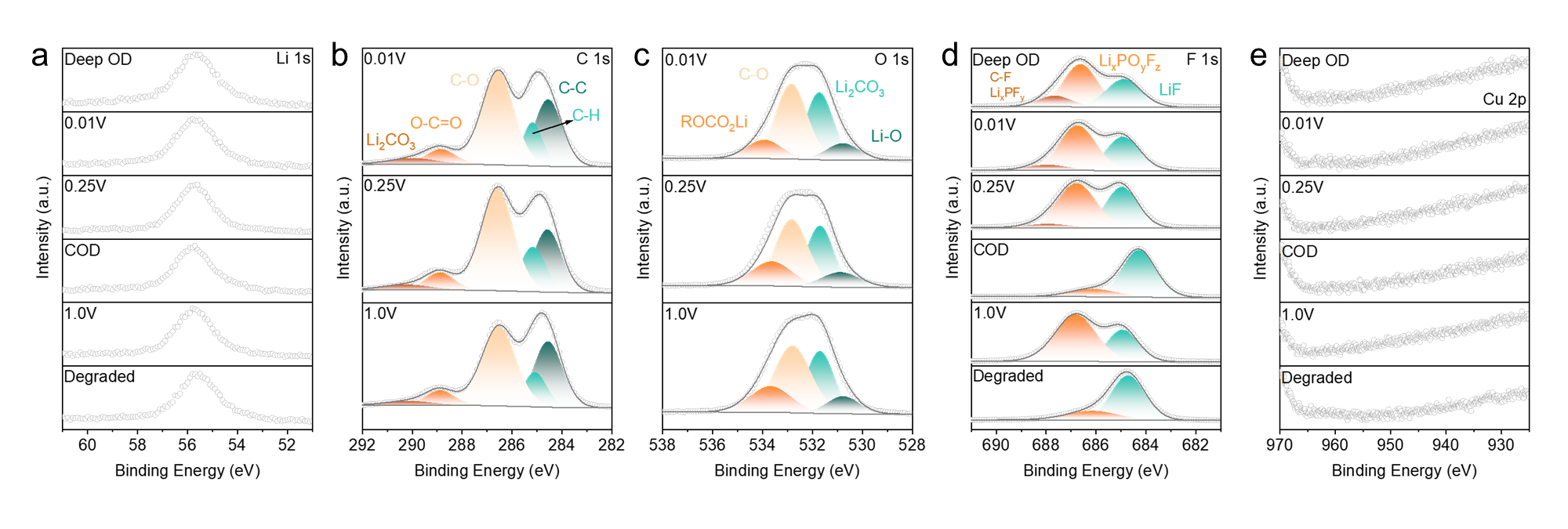


**Figure S14.** XPS analysis for graphite of a) Li 1s for all conditions; b) C 1s for 1.0 V, 0.25 V, and 0.01 V; c) O 1s for 1.0 V, 0.25 V, and 0.01 V; d) F 1s for all conditions; e) Cu 2p for all conditions.


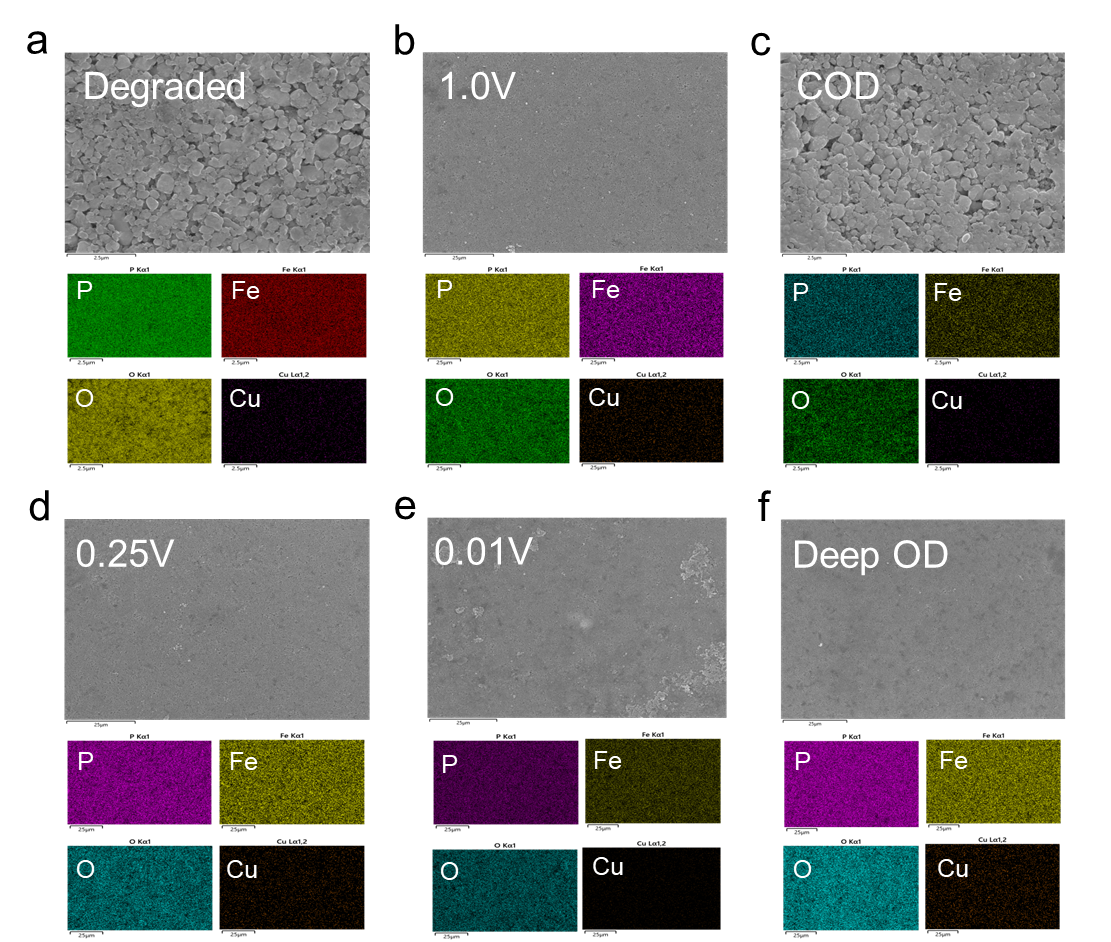


**Figure S15.** SEM image with EDS mapping for LFP in different discharge voltage from a) Degraded, b) 1.0 V, c) COD, d) 0.25 V, e) 0.01 V, to f) Deep OD.


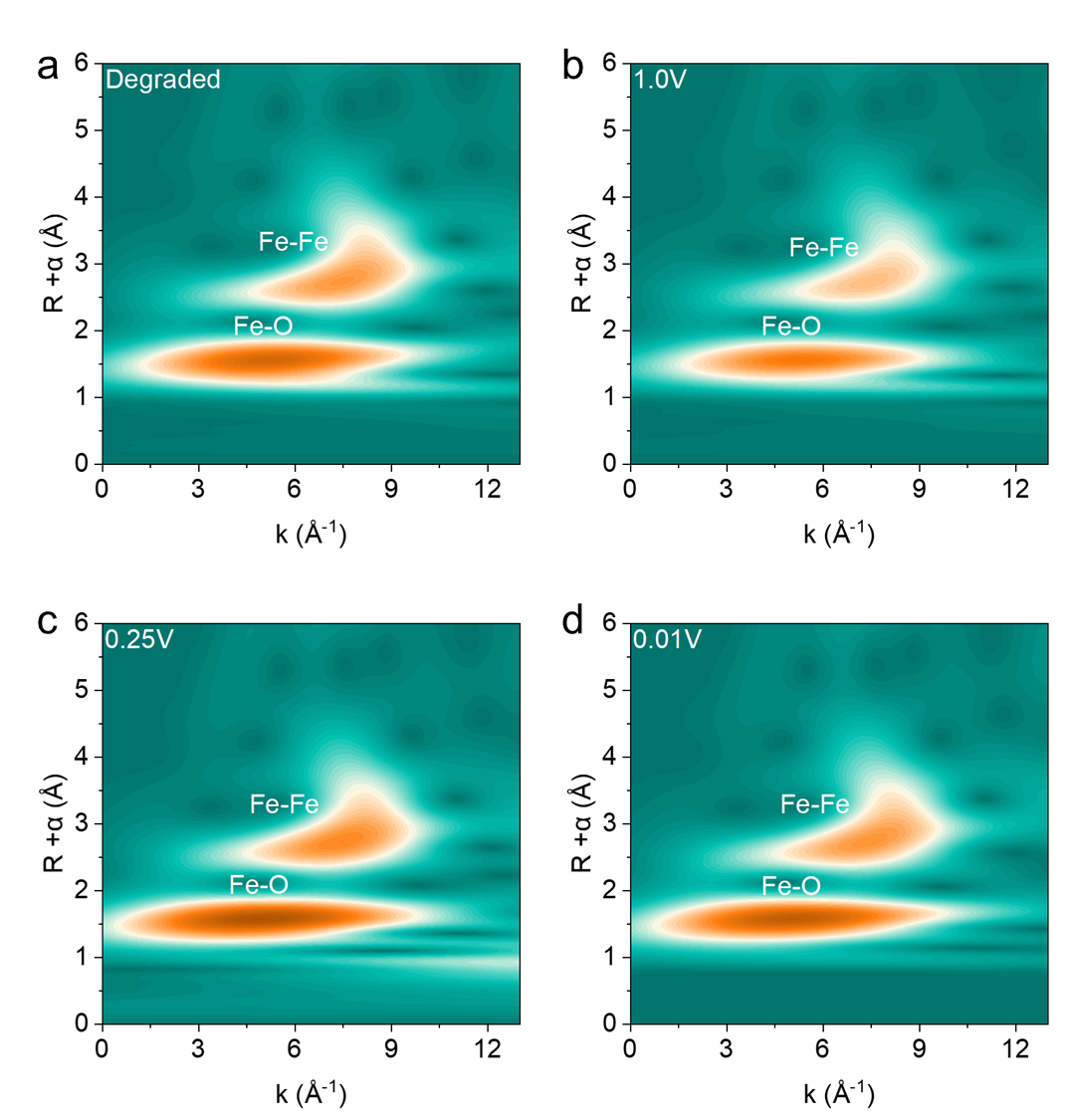


**Figure S16.** Wavelet transform of the EXAFS spectra for LFP in a) Degraded, b) 1.0 V, c) 0.25 V, and d) 0.01 V.


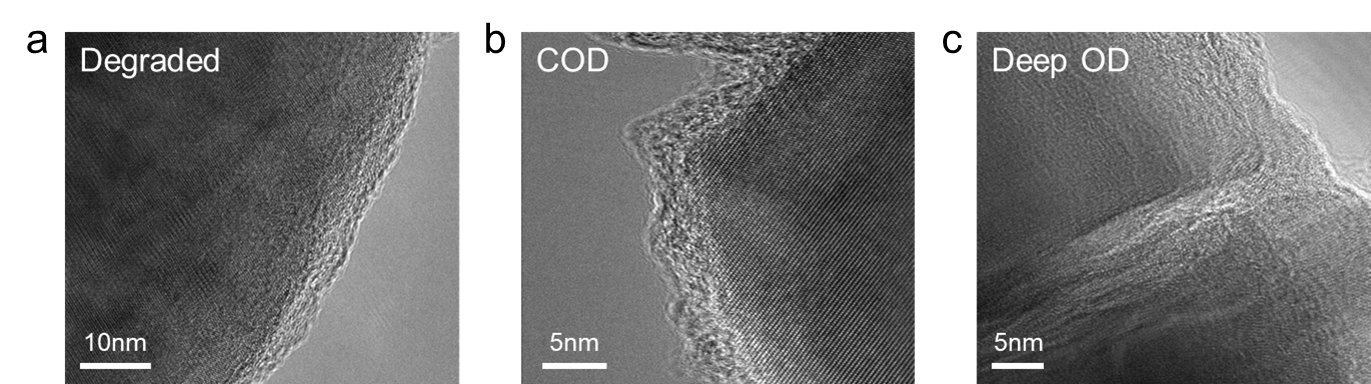


**Figure S17.** TEM images for LFP at different discharge voltages, a) Degraded, b) COD, and c) Deep OD.


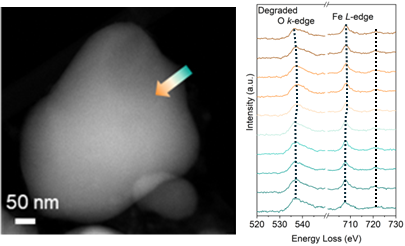


**Figure S18.** HAADF–STEM image and EELS line scan results across the interface showing the Fe L-edge and O K-edge spectra of Degraded.


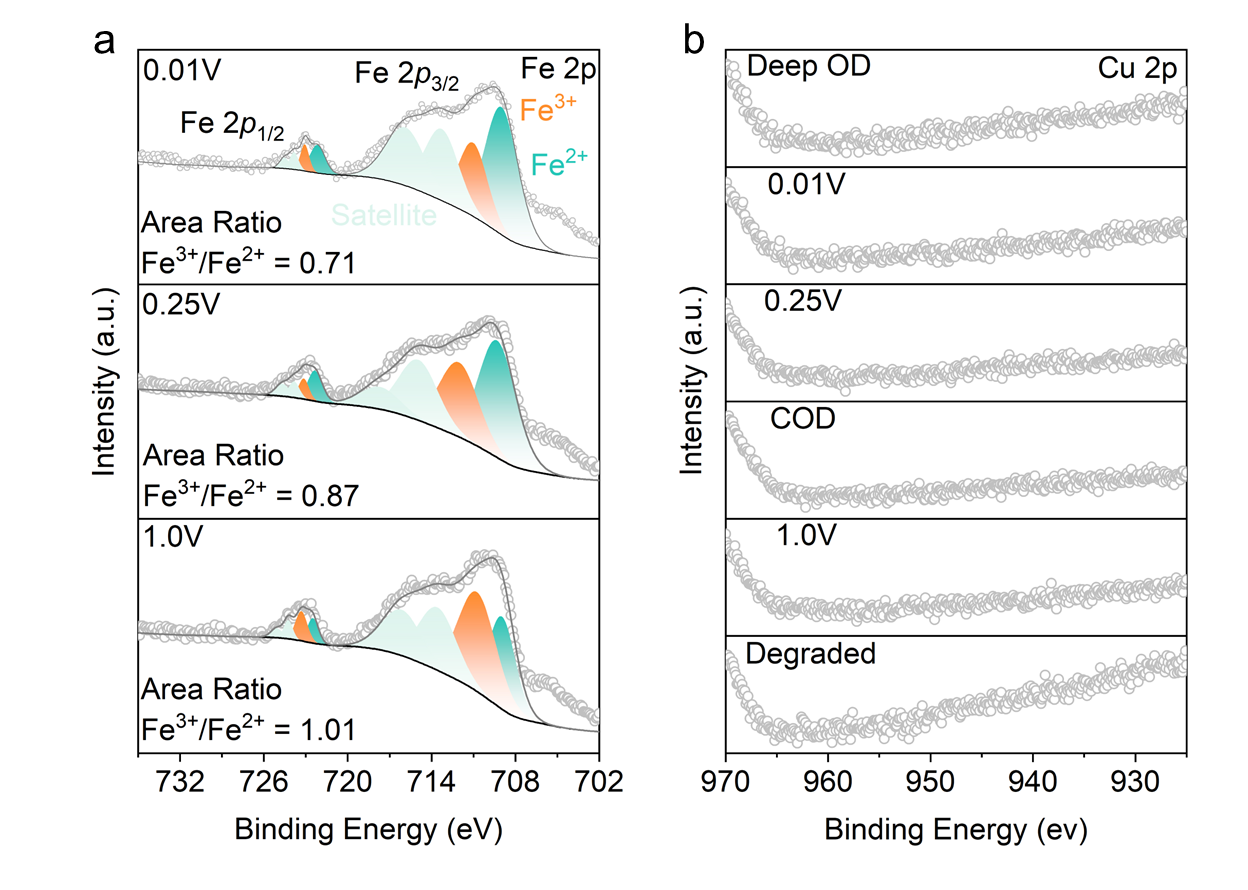


**Figure S19.** XPS analysis for LFP of a) Fe 2p for 1.0 V, 0.25 V and 0.01V; b) Cu 2p for Degraded, 1.0 V, COD, 0.25 V, 0.01 V and Deep OD.


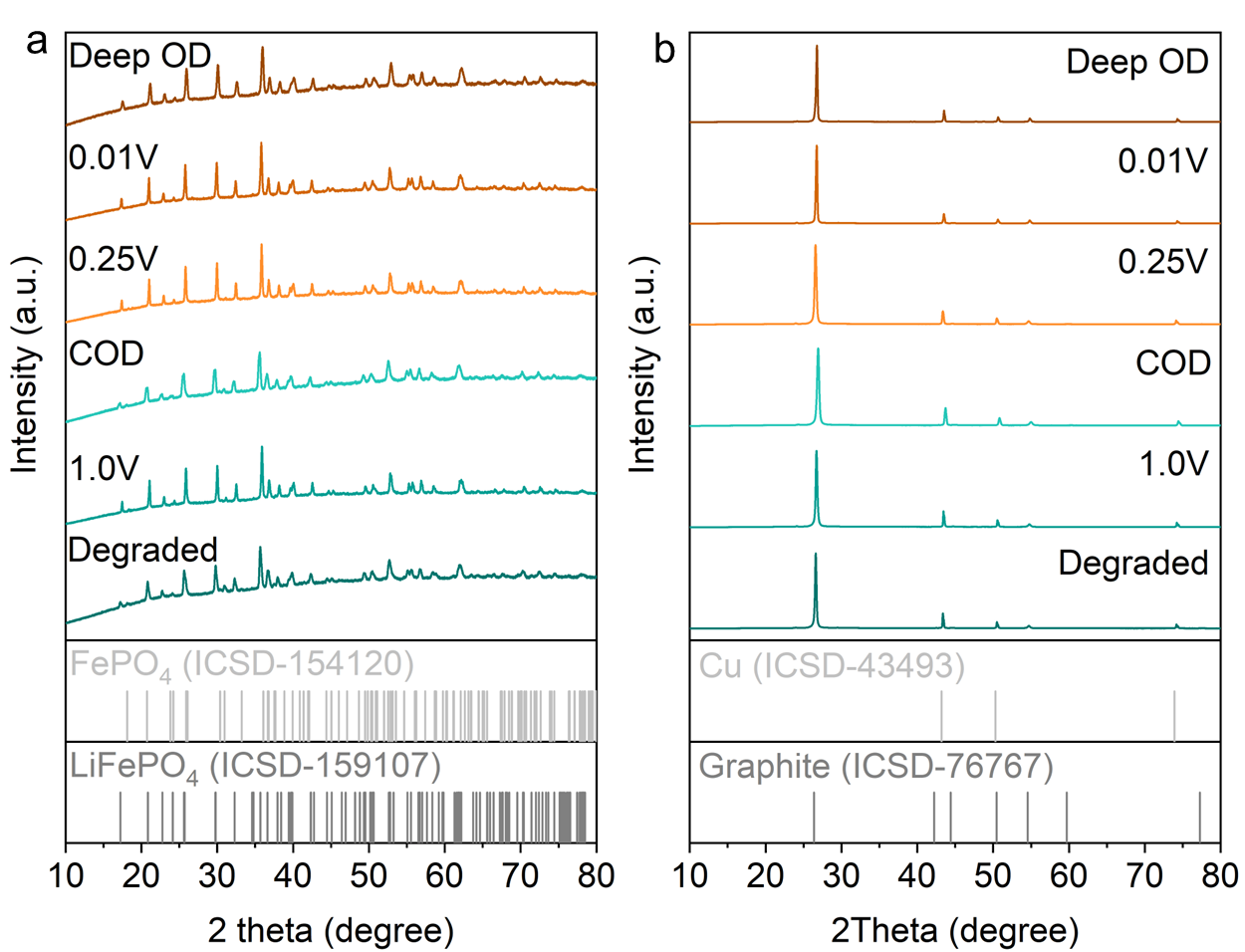


**Figure S20.** XRD patterns of LFP under different overdischarge conditions from full cell with a) LFP and b) graphite.


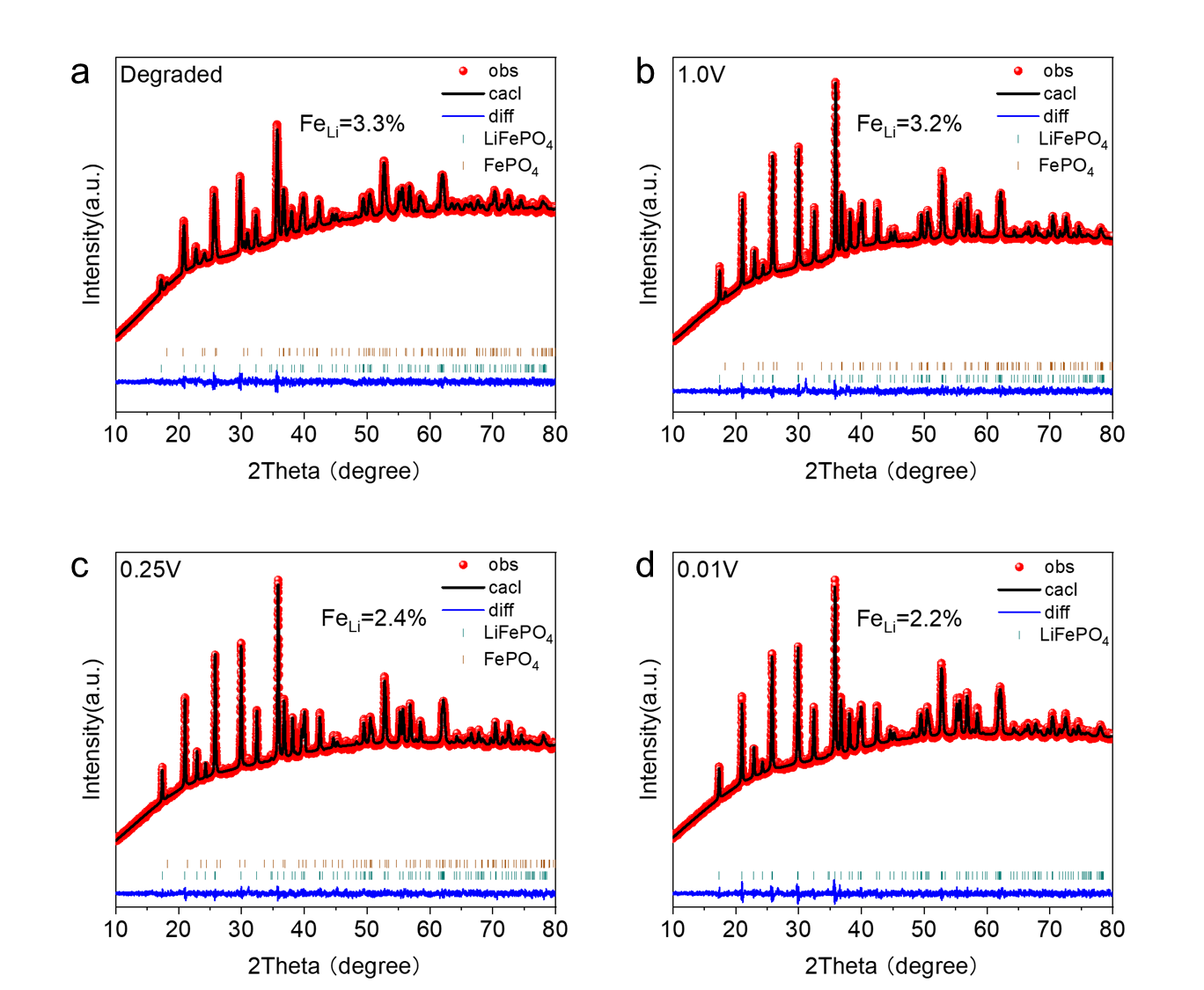


**Figure S21.** Rietveld refinements from ex-situ XRD patterns of a) Degraded, b) 1.0 V, c) 0.25 V, and d) 0.01 V.

# **Supplemental Tables**

**Table S1.** Lithium ion diffusion coefficients at different scanning rates of the Degraded and COD cells (De-: delithiation, Li-: lithiation).

| Scanning Rates (mV s^-1^) |  | 0.1 | 0.2 | 0.4 | 0.6 | 0.8 | 1.0 | Average |
| --- | --- | --- | --- | --- | --- | --- | --- | --- |
| Degraded (10^-11^ cm^2^ s^-1^) | De- | 1.3 | 1.01 | 0.76 | 0.63 | 0.53 | 0.44 | 0.77 |
|  | Li- | 1.27 | 0.95 | 0.68 | 0.53 | 0.43 | 0.36 | 0.7 |
| COD (10^-11^ cm^2^ s^-1^) | De- | 1.17 | 0.96 | 0.71 | 0.61 | 0.52 | 0.45 | 0.74 |
|  | Li- | 1.21 | 0.92 | 0.67 | 0.52 | 0.43 | 0.36 | 0.69 |

**Table S2.** Elemental analysis of graphite with different discharge conditions in EDS mapping.

|  | C (%) | O (%) | F (%) | Cu (%) |
| --- | --- | --- | --- | --- |
| Degraded | 92.12 | 5.73 | 2.06 | 0.09 |
| 1.0 V | 96.7 | 1.78 | 1.44 | 0.06 |
| COD | 93.49 | 5.31 | 1.11 | 0.09 |
| 0.25 V | 95.68 | 2.55 | 1.72 | 0.05 |
| 0.01 V | 93.99 | 2.55 | 3.32 | 0.13 |
| Deep OD | 81.26 | 4.57 | 14.02 | 0.12 |

**Table S3.** Rietveld refinement results of XRD of Degraded LFP at 2.5 V. Phase 1 LiFePO_4_: Space group: *Pnma*, a = 10.32549 Å, b = 6.00565 Å, c = 4.68941 Å. Phase 2 FePO_4_: Space group: *Pnma*, a = 9.81300 Å, b = 5.78433 Å, c = 4.77609 Å. R_p_ = 0.80%, R_wp_ = 1.07%

| LiFePO_4_ | | | | | | FePO_4_ | | | |
| --- | --- | --- | --- | --- | --- | --- | --- | --- | --- |
| Atom | Site | x | y | z | Occupancy | x | y | z | Occupancy |
| Li 1 | 4a | 0 | 0 | 0 | 0.967 | N/A | | | |
| Fe 1 | 4c | 0.283201 | 0.25000 | 0.975622 | 0.967 | 0.278312 | 0.25000 | 0.957337 | 0.964 |
| P | 4c | 0.098068 | 0.25000 | 0.435921 | 1 | 0.101537 | 0.25000 | 0.380533 | 1 |
| O 1 | 4c | 0.102061 | 0.25000 | 0.759652 | 1 | 0.126207 | 0.25000 | 0.736079 | 1 |
| O 2 | 4c | 0.457745 | 0.25000 | 0.198610 | 1 | 0.438292 | 0.25000 | 0.153894 | 1 |
| O 3 | 8d | 0.194743 | 0.051996 | 0.290666 | 1 | 0.155482 | 0.048004 | 0.245180 | 1 |
| Li 2 | 4c | 0.283201 | 0.25000 | 0.975622 | 0.033 | N/A | | | |
| Fe 2 | 4a | 0 | 0 | 0 | 0.033 | 0 | 0 | 0 | 0.036 |

**Table S4.** Rietveld refinement results of XRD of Degraded LFP at 1.0 V. Phase 1 LiFePO_4_: Space group: *Pnma*, a = 10.33438 Å, b = 6.00972 Å, c = 4.69437 Å. Phase 2 FePO_4_: Space group: *Pnma*, a = 9.81988 Å, b = 6.02078 Å, c = 4.68703 Å. R_p_ = 0.87%, R_wp_ = 1.15%

| LiFePO_4_ | | | | | | FePO_4_ | | | | | |
| --- | --- | --- | --- | --- | --- | --- | --- | --- | --- | --- | --- |
| Atom | Site | x | y | z | Occupancy | x | | y | | Z | Occupancy |
| Li 1 | 4a | 0 | 0 | 0 | 0.968 | N/A | | | | | |
| Fe 1 | 4c | 0.282690 | 0.25000 | 0.974309 | 0.968 | 0.266230 | 0.25000 | | 0.960056 | | 0.985 |
| P | 4c | 0.097916 | 0.25000 | 0.413454 | 1 | 0.116991 | 0.25000 | | 0.418181 | | 1 |
| O 1 | 4c | 0.092967 | 0.25000 | 0.755122 | 1 | 0.114096 | 0.25000 | | 0.702795 | | 1 |
| O 2 | 4c | 0.459471 | 0.25000 | 0.223514 | 1 | 0.437673 | 0.25000 | | 0.136145 | | 1 |
| O 3 | 8d | 0.178758 | 0.045581 | 0.291172 | 1 | 0.152403 | 0.046710 | | 0.247083 | | 1 |
| Li 2 | 4c | 0.282690 | 0.25000 | 0.974309 | 0.032 | N/A | | | | | |
| Fe 2 | 4a | 0 | 0 | 0 | 0.032 | 0 | 0 | | 0 | | 0.015 |

**Table S5.** Rietveld refinement results of XRD of Degraded LFP at COD. Phase 1 LiFePO_4_: Space group: *Pnma*, a = 10.31807 Å, b = 6.00200 Å, c = 4.68665 Å. Phase 2 FePO_4_: Space group: *Pnma*, a = 9.81927 Å, b = 5.78094 Å, c = 4.77884 Å. R_p_ = 0.98%, R_wp_ = 1.29%

| LiFePO_4_ | | | | | | FePO_4_ | | | |
| --- | --- | --- | --- | --- | --- | --- | --- | --- | --- |
| Atom | Site | x | y | z | Occupancy | x | y | z | Occupancy |
| Li 1 | 4a | 0 | 0 | 0 | 0.974 | N/A | | | |
| Fe 1 | 4c | 0.283918 | 0.25000 | 0.986032 | 0.974 | 0.271458 | 0.25000 | 0.952096 | 0.995 |
| P | 4c | 0.096266 | 0.25000 | 0.415407 | 1 | 0.124447 | 0.25000 | 0.437286 | 1 |
| O 1 | 4c | 0.098381 | 0.25000 | 0.769057 | 1 | 0.125476 | 0.25000 | 0.427782 | 1 |
| O 2 | 4c | 0.457485 | 0.25000 | 0.246537 | 1 | 0.441572 | 0.25000 | 0.165556 | 1 |
| O 3 | 8d | 0.184423 | 0.023890 | 0.27149 | 1 | 0.152981 | 0.052255 | 0.206181 | 1 |
| Li 2 | 4c | 0.283918 | 0.25000 | 0.986032 | 0.026 | N/A | | | |
| Fe 2 | 4a | 0 | 0 | 0 | 0.026 | 0 | 0 | 0 | 0.005 |

**Table S6.** Rietveld refinement results of XRD of Degraded LFP at 0.25 V. Phase 1 LiFePO_4_: Space group: *Pnma*, a = 10.33469 Å, b = 6.00971 Å, c = 4.69368 Å. Phase 2 FePO_4_: Space group: *Pnma*, a = 9.82947 Å, b = 5.79000 Å, c = 4.78300 Å. R_p_ = 0.88%, R_wp_ = 1.13%

| LiFePO_4_ | | | | | | FePO_4_ | | | |
| --- | --- | --- | --- | --- | --- | --- | --- | --- | --- |
| Atom | Site | x | y | z | Occupancy | x | y | z | Occupancy |
| Li 1 | 4a | 0 | 0 | 0 | 0.976 | N/A | | | |
| Fe 1 | 4c | 0.282170 | 0.25000 | 0.972958 | 0.976 | 0.253976 | 0.25000 | 0.948210 | 0.984 |
| P | 4c | 0.098250 | 0.25000 | 0.426754 | 1 | 0.145880 | 0.25000 | 0.358504 | 1 |
| O 1 | 4c | 0.100370 | 0.25000 | 0.754686 | 1 | 0.198278 | 0.25000 | 0.709656 | 1 |
| O 2 | 4c | 0.462669 | 0.25000 | 0.209907 | 1 | 0.432258 | 0.25000 | 0.167314 | 1 |
| O 3 | 8d | 0.188239 | 0.045360 | 0.288522 | 1 | 0.250791 | 0.068447 | 0.234630 | 1 |
| Li 2 | 4c | 0.282170 | 0.25000 | 0.972958 | 0.024 | N/A | | | |
| Fe 2 | 4a | 0 | 0 | 0 | 0.024 | 0 | 0 | 0 | 0.016 |

**Table S7.** Rietveld refinement results of XRD of Degraded LFP at 0.01 V. Phase 1 LiFePO_4_: Space group: *Pnma,* a = 10.33376 Å, b = 6.01150 Å, c = 4.69331 Å. R_p_ = 0.90%, R_wp_ = 1.21%

| LiFePO_4_ | | | | | |
| --- | --- | --- | --- | --- | --- |
| Atom | Site | x | y | z | Occupancy |
| Li 1 | 4a | 0 | 0 | 0 | 0.978 |
| Fe 1 | 4c | 0.284000 | 0.25000 | 0.974509 | 0.978 |
| P | 4c | 0.097262 | 0.25000 | 0.426685 | 1 |
| O 1 | 4c | 0.101457 | 0.25000 | 0.756703 | 1 |
| O 2 | 4c | 0.467460 | 0.25000 | 0.198988 | 1 |
| O 3 | 8d | 0.181843 | 0.046372 | 0.292816 | 1 |
| Li 2 | 4c | 0.284000 | 0.25000 | 0.974509 | 0.022 |
| Fe 2 | 4a | 0 | 0 | 0 | 0.022 |

**Table S8.** Rietveld refinement results of XRD of Deep OD LFP. Phase 1 LiFePO_4_: Space group: *Pnma*, a = 10.34267 Å, b = 6.01463 Å, c = 4.69671 Å. R_p_ = 0.81%, R_wp_ = 1.02%

| LiFePO_4_ | | | | | |
| --- | --- | --- | --- | --- | --- |
| Atom | Site | x | y | z | Occupancy |
| Li 1 | 4a | 0 | 0 | 0 | 0.978 |
| Fe 1 | 4c | 0.283692 | 0.25000 | 0.974252 | 0.978 |
| P | 4c | 0.096719 | 0.25000 | 0.424406 | 1 |
| O 1 | 4c | 0.100063 | 0.25000 | 0.750601 | 1 |
| O 2 | 4c | 0.466802 | 0.25000 | 0.200013 | 1 |
| O 3 | 8d | 0.176869 | 0.045764 | 0.292437 | 1 |
| Li 2 | 4c | 0.283692 | 0.25000 | 0.974252 | 0.022 |
| Fe 2 | 4a | 0 | 0 | 0 | 0.022 |

**Table S9.** Representative non-destructive methods reported for spent battery regeneration.

| No | Pathway | Method | Recovery % | Ref |
| --- | --- | --- | --- | --- |
| 1 | Non-invasive | Overdischarge | 9.56 | This work |
| 2 | Non-invasive | Overdischarge | 4.04 | [1] |
| 3 | Non-disassemble | Reagent Injection | 4 | [2] |
| 4 | Non-disassemble | Reagent Injection | 5.89 | [3] |
| 5 | Non-disassemble | Reagent Injection | 14.6 | [4] |
| 6 | Non-invasive | Magneto-electrochemical | 10.02 | [5] |

**Table S10.** Materials requirements (kg) to recycle 1 kg of spent batteries through different recycling technologies.

| Material Inputs (kg) | Pyrometallurgy | | Hydrometallurgy | Direct Recycling | Current Research |
| --- | --- | --- | --- | --- | --- |
| Hydrochloric Acid | | 0.21 | 0.012 |  |  |
| Hydrogen Peroxide | | 0.06 | 0.366 |  |  |
| Sodium | |  | 0.561 |  |  |
| Limestone  Sand | | 0.3 |  |  |  |
| Sulfuric Acid | | 0.15 |  |  |  |
| Soda Ash | |  | 1.078 |  |  |
| Citric Acid | |  | 0.02 | 0.007 |  |
| Lithium Hydroxide | |  |  | 0.022 |  |
| Lithium Carbonate | |  |  | 0.003 |  |

**Table S11.** Energy requirements (MJ) to recycle 1 kg of spent battery through different recycling technologies.

|  | Pyrometallurgy | | Hydrometallurgy | Direct Recycling | Current Research |
| --- | --- | --- | --- | --- | --- |
| Diesel | | 0.6 | 0.6 | 0.6 |  |
| Natural gas | |  | 2.5 |  |  |
| Electricity | | 4.68 | 0.13 | 0.29 | 1.3 |

**Table S12.** Life-cycle environmental impacts of different recycling methods.

|  | Pyrometallurgy | | Hydrometallurgy | | Direct Recycling | Current Research |
| --- | --- | --- | --- | --- | --- | --- |
| Total Energy use in MJ kg^-1^ cell in recycled | | | | | | |
| Total Energy | | 15.710 | | 23.337 | 9.006 | 3.17 |
| Fossil Fuels | | 13.799 | | 21.630 | 8.593 | 2.11 |
| Coal | | 6.096 | | 5.977 | 5.521 | 1.06 |
| Natural gas | | 9.596 | | 16.844 | 4.209 | 1.06 |
| Petroleum | | 3.307 | | 4.008 | 4.062 | 0 |
| Total Emission in g kg^-1^ cell in recycled | | | | | | |
| VOC | | 0.178 | | 0.275 | 0.161 | 0.019 |
| CO | | 0.701 | | 1.094 | 0.604 | 0.067 |
| NO_X_ | | 1.368 | | 2.089 | 1.416 | 0.121 |
| PM 10 | | 0.114 | | 0.157 | 0.134 | 0.018 |
| PM 2.5 | | 0.081 | | 0.120 | 0.105 | 0.010 |
| SO_x_ | | 0.450 | | 2.090 | 0.567 | 0.107 |
| BC | | 0.025 | | 0.031 | 0.03 | 0.001 |
| OC | | 0.021 | | 0.036 | 0.026 | 0.003 |
| CH_4_ | | 2.233 | | 3.665 | 1.266 | 0.334 |
| N_2_O | | 0.022 | | 0.034 | 0.015 | 0.003 |
| CO_2_ | | 2,098 | | 1,644 | 897 | 157 |
| CO_2_ (w/C in VOC & CO) | | 2,100 | | 1,647 | 899 | 158 |
| GHGs | | 2,173 | | 1,765 | 941 | 168 |

**References**

[1] X. Zhao, H. Chen, H. Wu, et al., “Nondisassembly Repair of Degraded LiFePO_4_ Cells via Lithium Restoration from the Solid Electrolyte Interphase,” *ACS Nano* 18 (2024): 21125-21134.

[2] N. Ogihara, K. Nagaya, H. Yamaguchi, et al., “Direct Capacity Regeneration for Spent Li-Ion Batteries,” *Joule* 8 (2024): 1364-1379.

[3] Y. Gao, H.-M. Zhang, J. Sun, “In-situ Capacity Regeneration of Degraded Lithium-Ion Batteries using Remanufacturing Remediator,” *Energy Storage Materials* 78 (2025): 104248.

[4] S. Chen, G. Wu, H. Jiang, et al., “External Li Supply Reshapes Li Deficiency and Lifetime Limit of Batteries,” *Nature* 638 (2025): 676-683.

[5] H. Gong, Y. Cao, B. Zhang, et al., “Noninvasive rejuvenation strategy of nickel-rich layered positive electrode for Li-ion battery through magneto-electrochemical synergistic activation,” *Nature Communications* 15 (2024): 10243.
